# Supplementary figures and images for: Genome-wide analysis of horizontal transfer in non-model wild species from a natural ecosystem reveals new insights into genetic exchange in plants
Source: PLoS Genet. 2023 Oct 19;19(10):e1010964. doi: 10.1371/journal.pgen.1010964 (PMC10586619; doi:10.1371/journal.pgen.1010964)

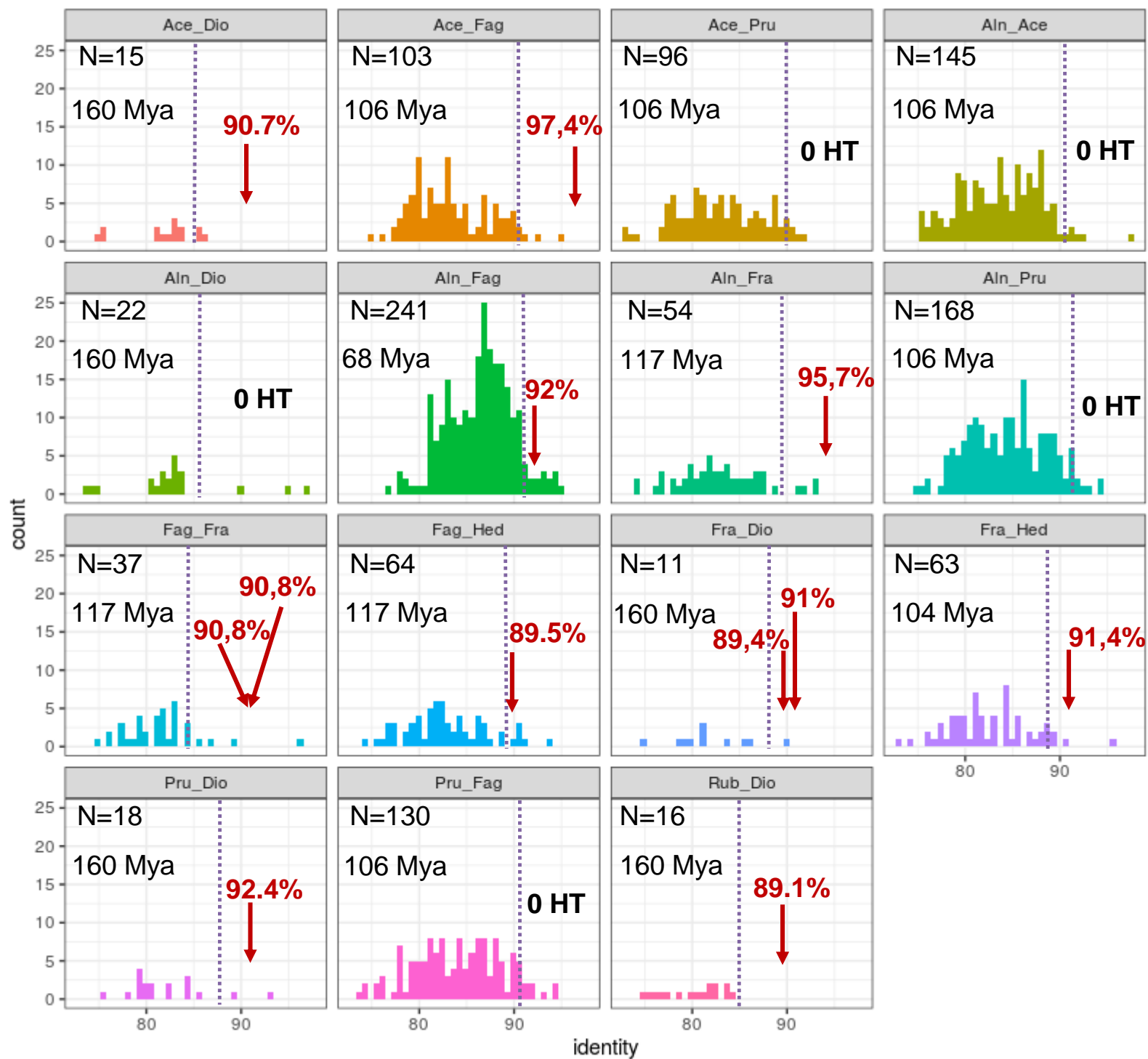

Supplement: S1 Fig — These sequence identities were obtained by Blastn alignment. N: corresponds to the total number of BUSCO genes that can be aligned at the nucleic level between each pair of species. HS threshold calculated by INTERCHANGE using the following formula: HS = (Q3+IQR/2), the inter-quartile range IQR = Q3-Q1 (Q1 and Q3 correspond to the first and third quartile respectively). The age in millions of years (Mya) represents the divergence time between species according to Timetree.org. Correspondence of species names: Ace: Acer monspessulanum, Aln: Alnus glutinosa, Bry: Bryonia dioica, Dio: Dioscorea communis, Fag: Fagus sylvatica, Fra: Fraxinus excelsior, Fom: Fomes fomentarius, Gen: Genista pilosa, Hed: Hedera helix, Her: Hericium clathroides, Lon: Lonicera periclymenum, Ple: Pleurotus ostreatus, Pru: Prunus avium, Rub: Rubus ulmifolius, Sal: Salvia sp, Sen: Senecio inaequidens, Sor: Sorbus aria. (PDF) [file pgen.1010964.s001.pdf]

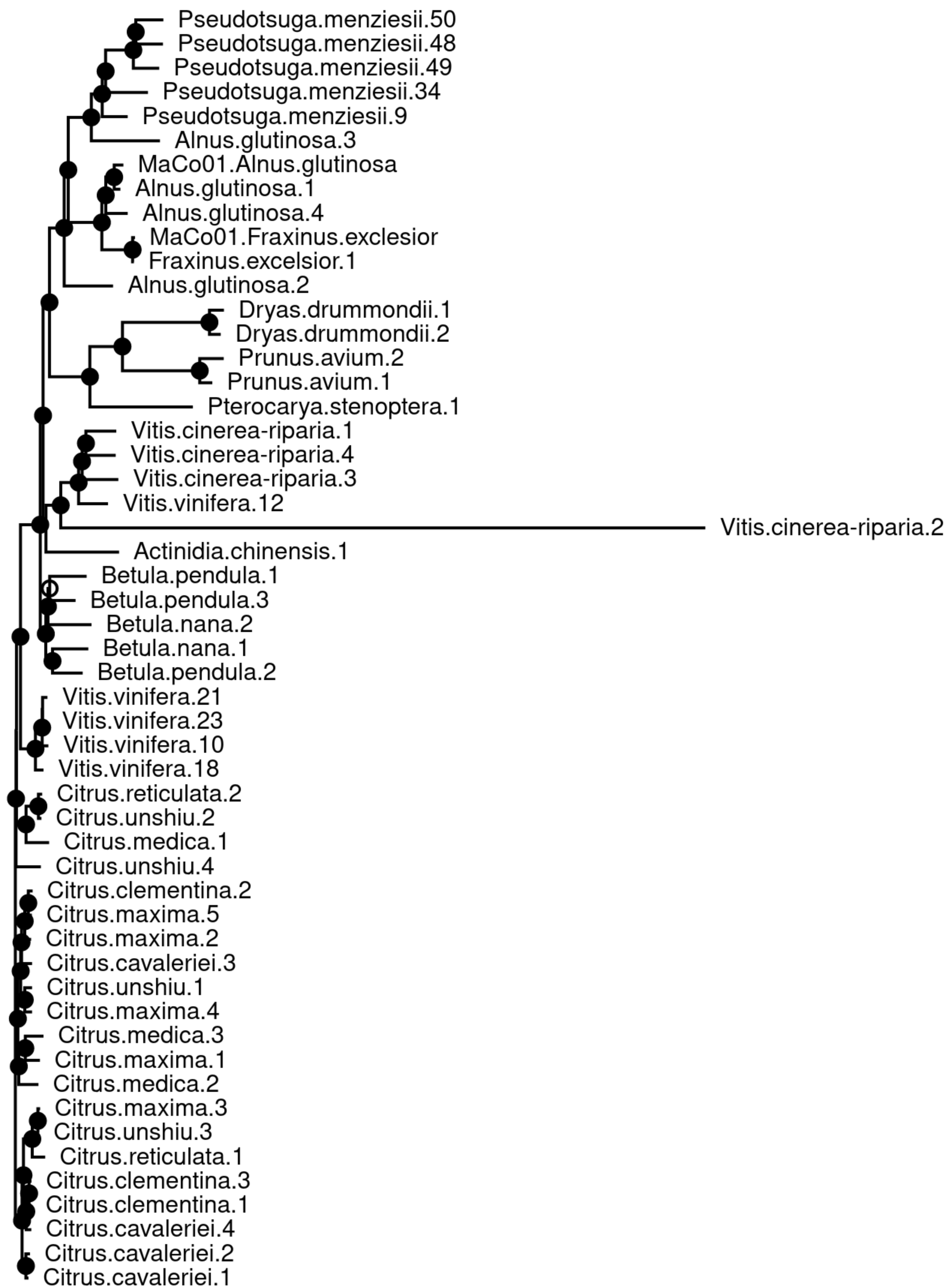

Supplement: S2 Fig — Nodes supported with boostrap values above 70% are indicated with a black dot. Nodes with bootstrap values under 70% are indicated with white dot. (PDF) [file pgen.1010964.s002.pdf]

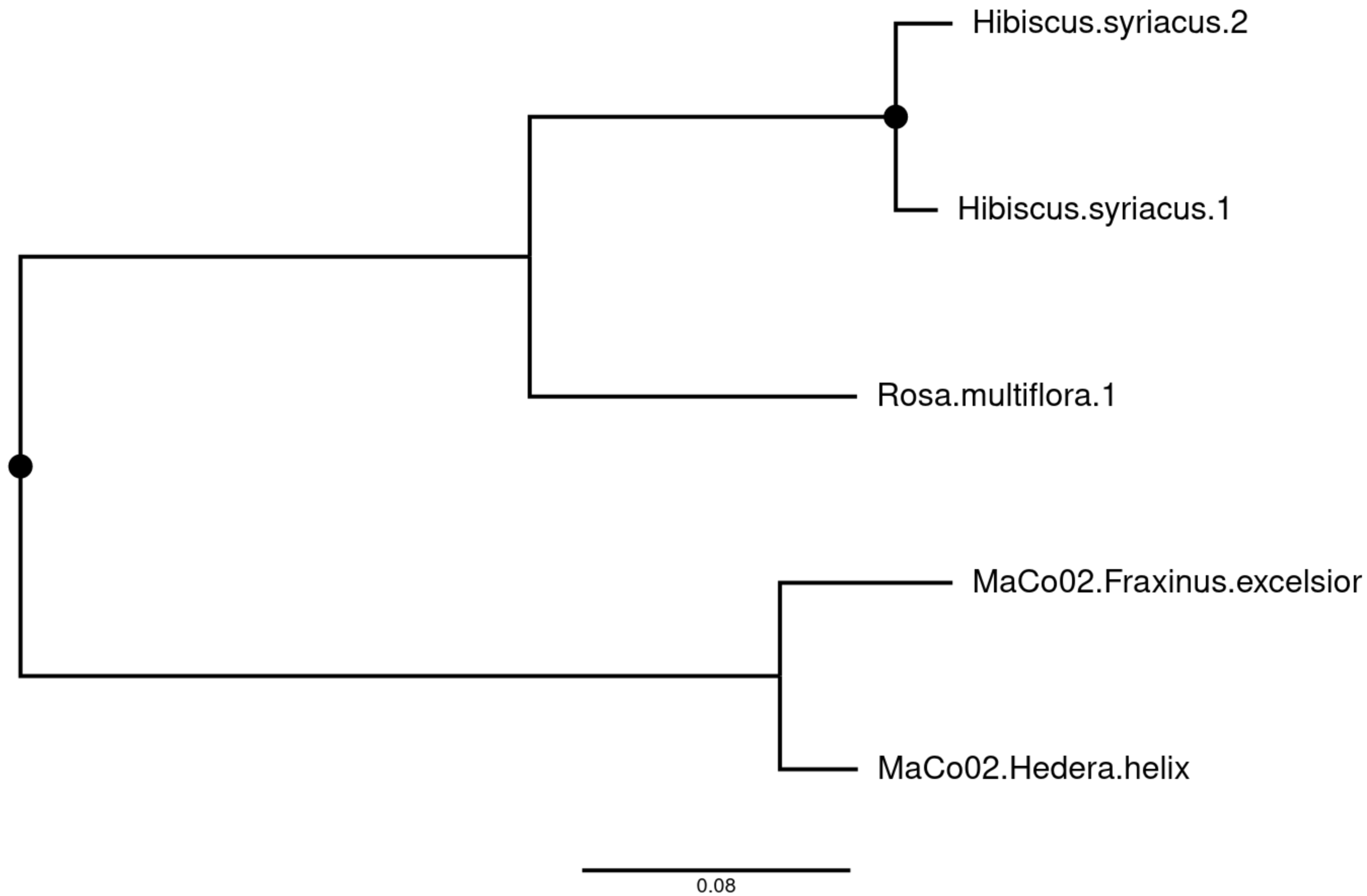

Supplement: S3 Fig — Nodes supported with boostrap values above 70% are indicated with a black dot. Nodes with bootstrap values under 70% are indicated with white dot. (PDF) [file pgen.1010964.s003.pdf]

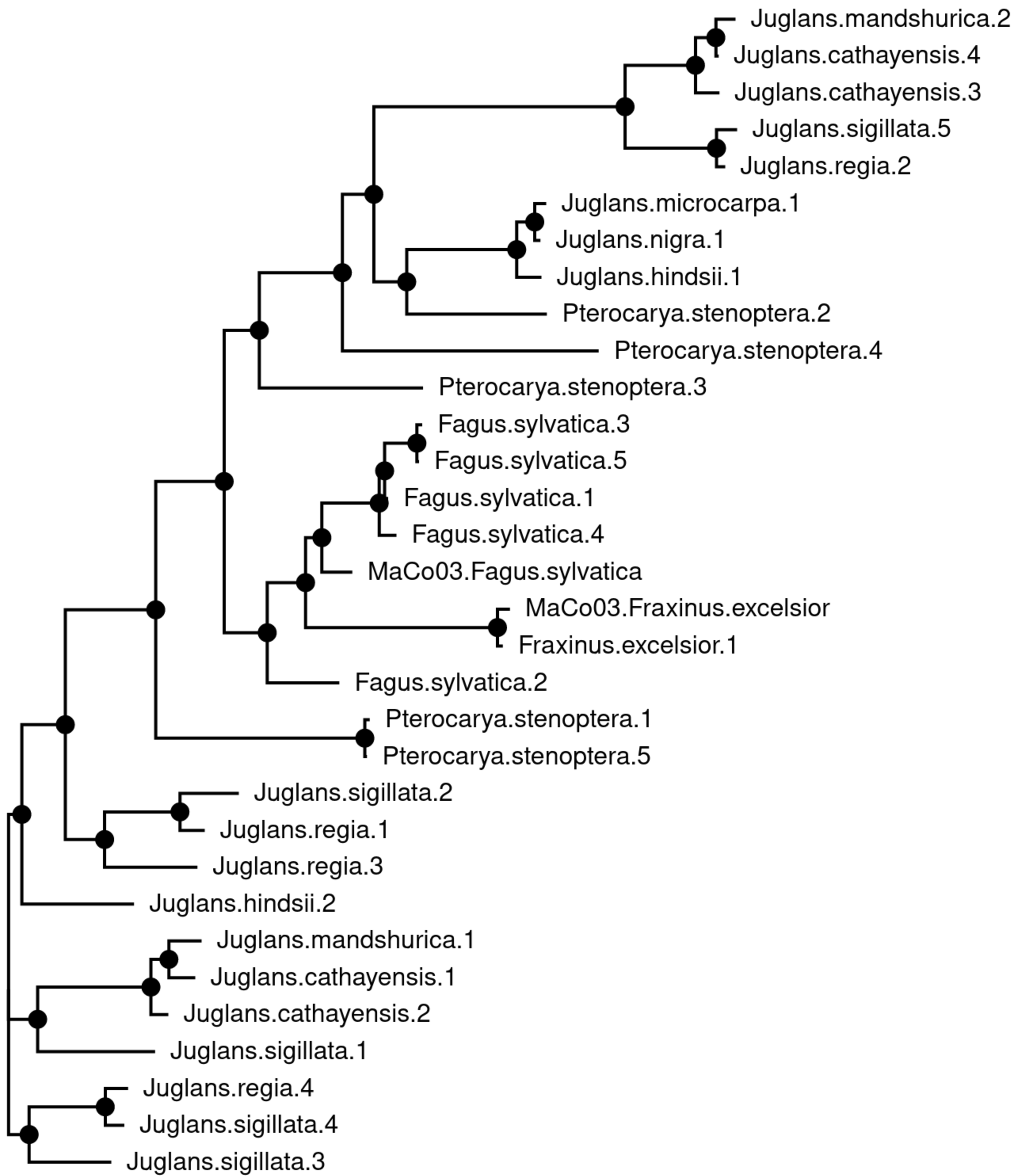

0.06

Supplement: S4 Fig — Nodes supported with boostrap values above 70% are indicated with a black dot. Nodes with bootstrap values under 70% are indicated with white dot. (PDF) [file pgen.1010964.s004.pdf]

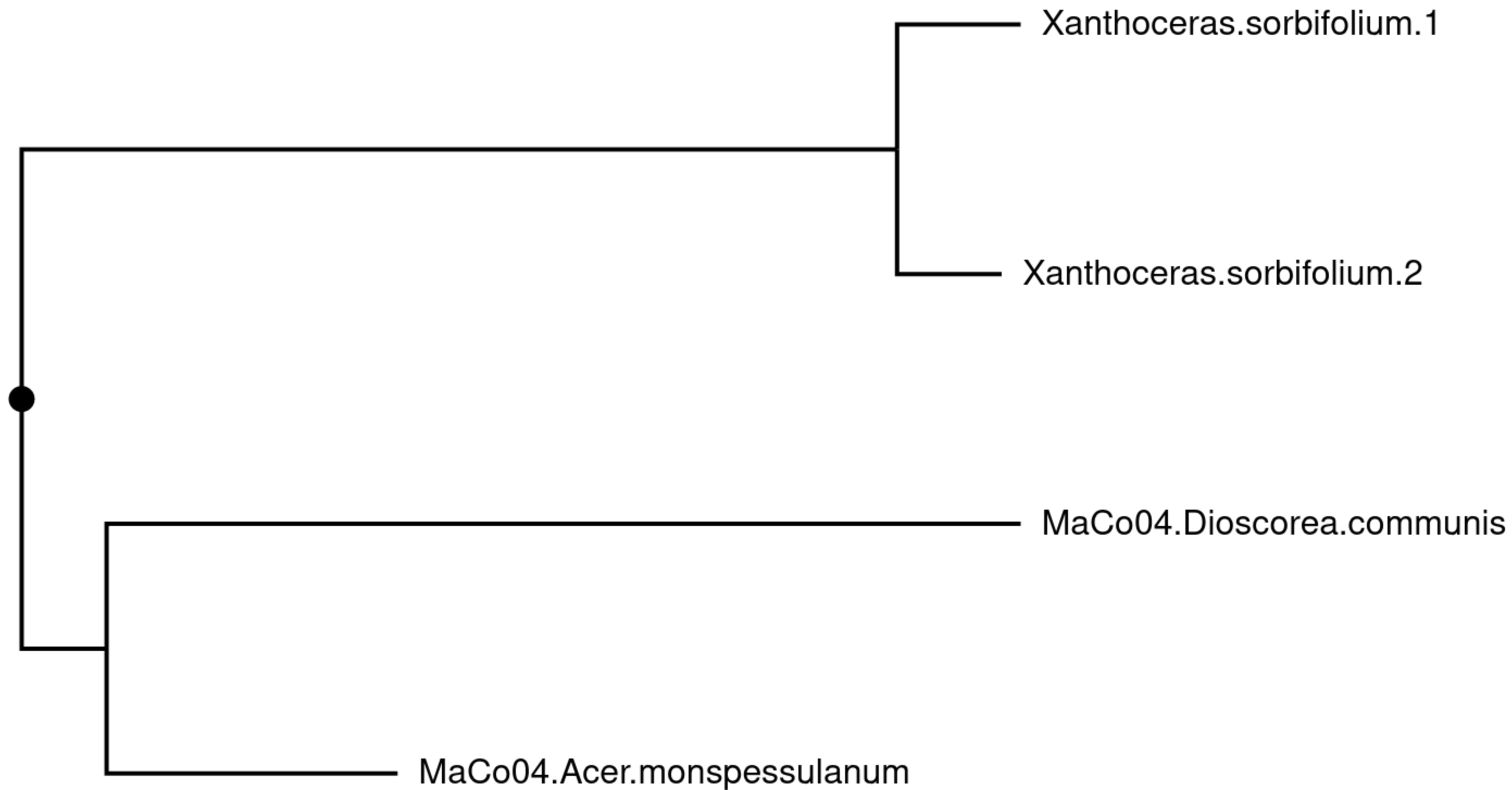

0.02

Supplement: S5 Fig — Nodes supported with boostrap values above 70% are indicated with a black dot. Nodes with bootstrap values under 70% are indicated with white dot. (PDF) [file pgen.1010964.s005.pdf]

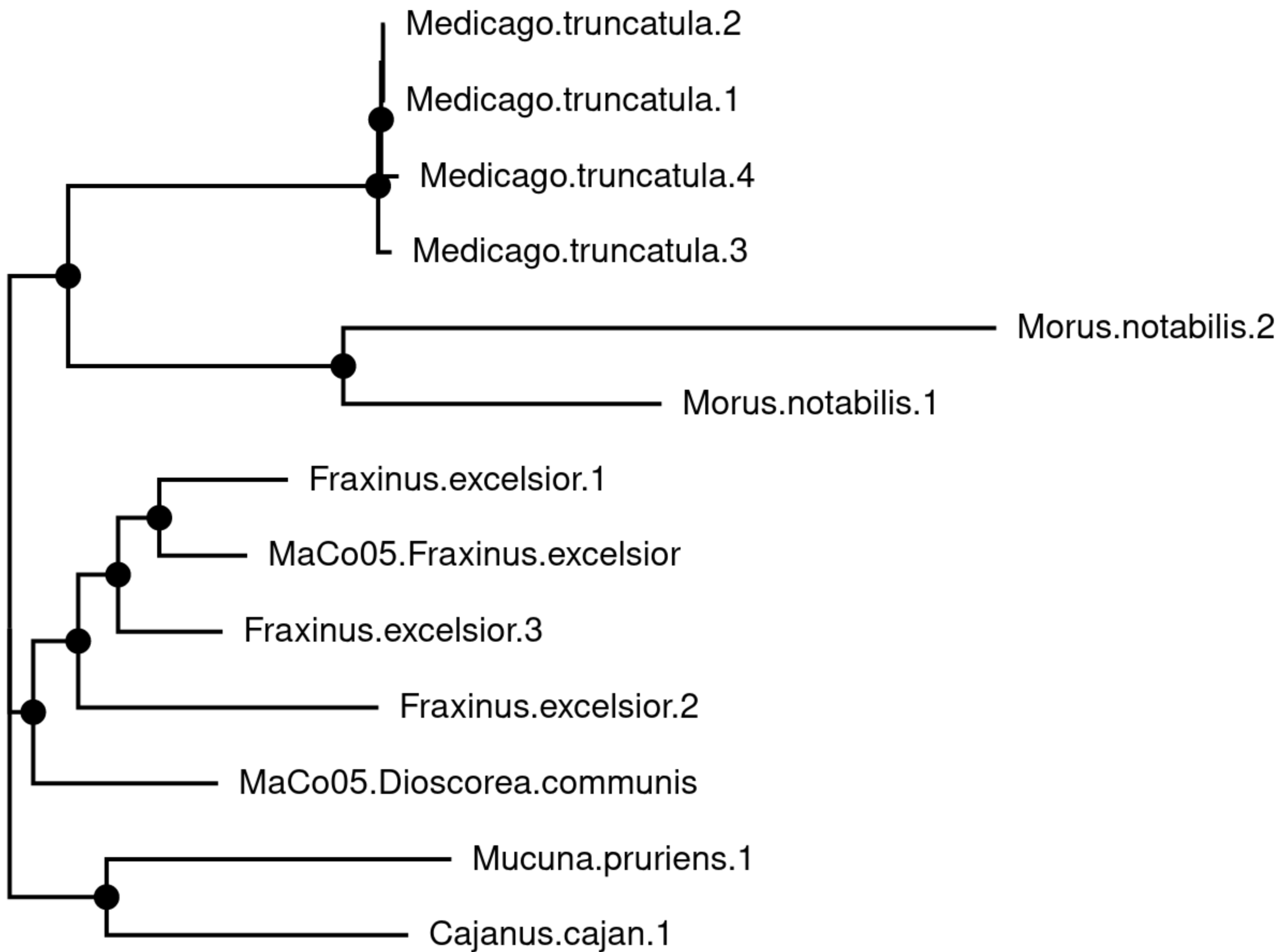

0.06

Supplement: S6 Fig — Nodes supported with boostrap values above 70% are indicated with a black dot. Nodes with bootstrap values under 70% are indicated with white dot. (PDF) [file pgen.1010964.s006.pdf]

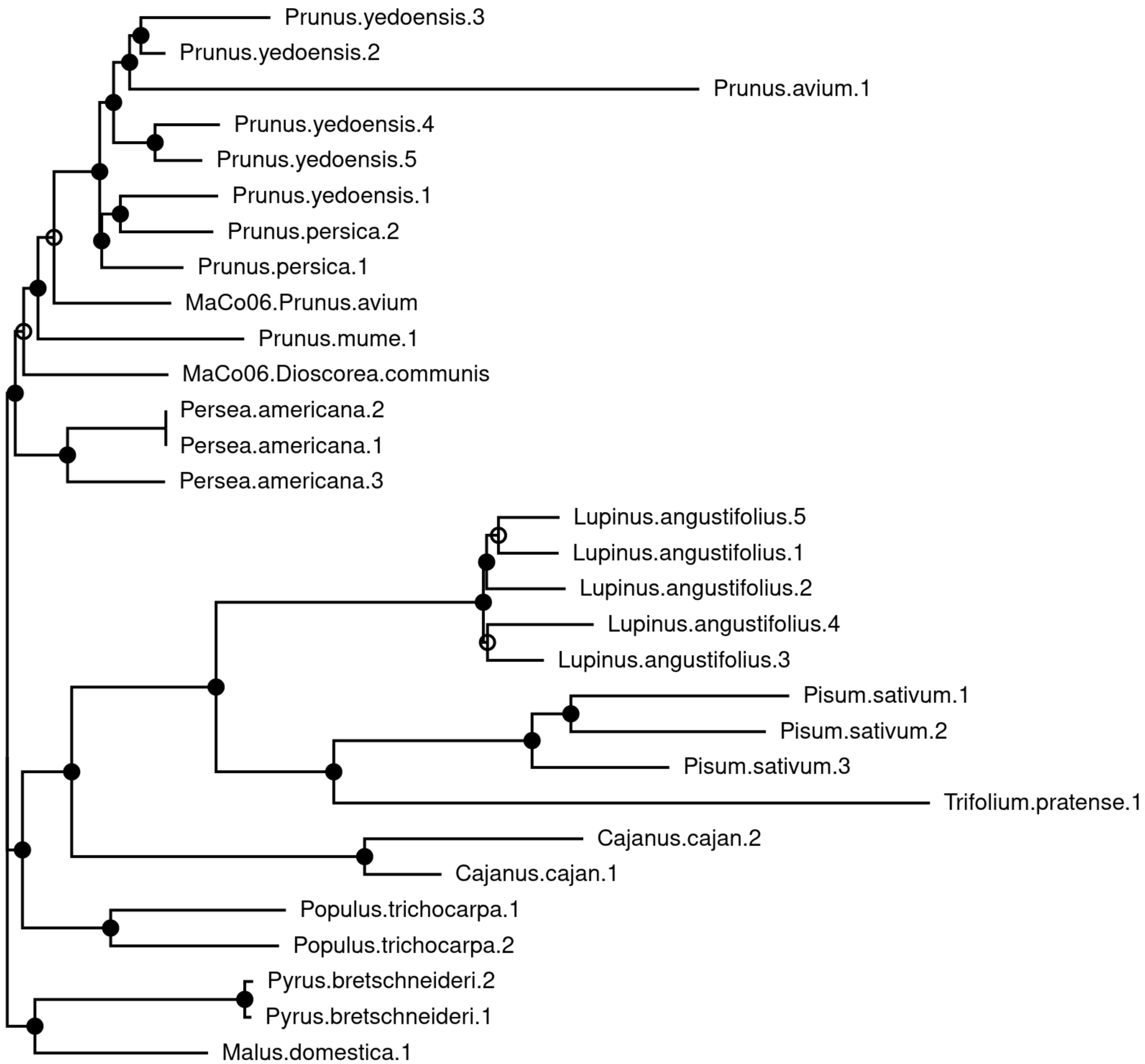

0.04

Supplement: S7 Fig — Nodes supported with boostrap values above 70% are indicated with a black dot. Nodes with bootstrap values under 70% are indicated with white dot. (PDF) [file pgen.1010964.s007.pdf]

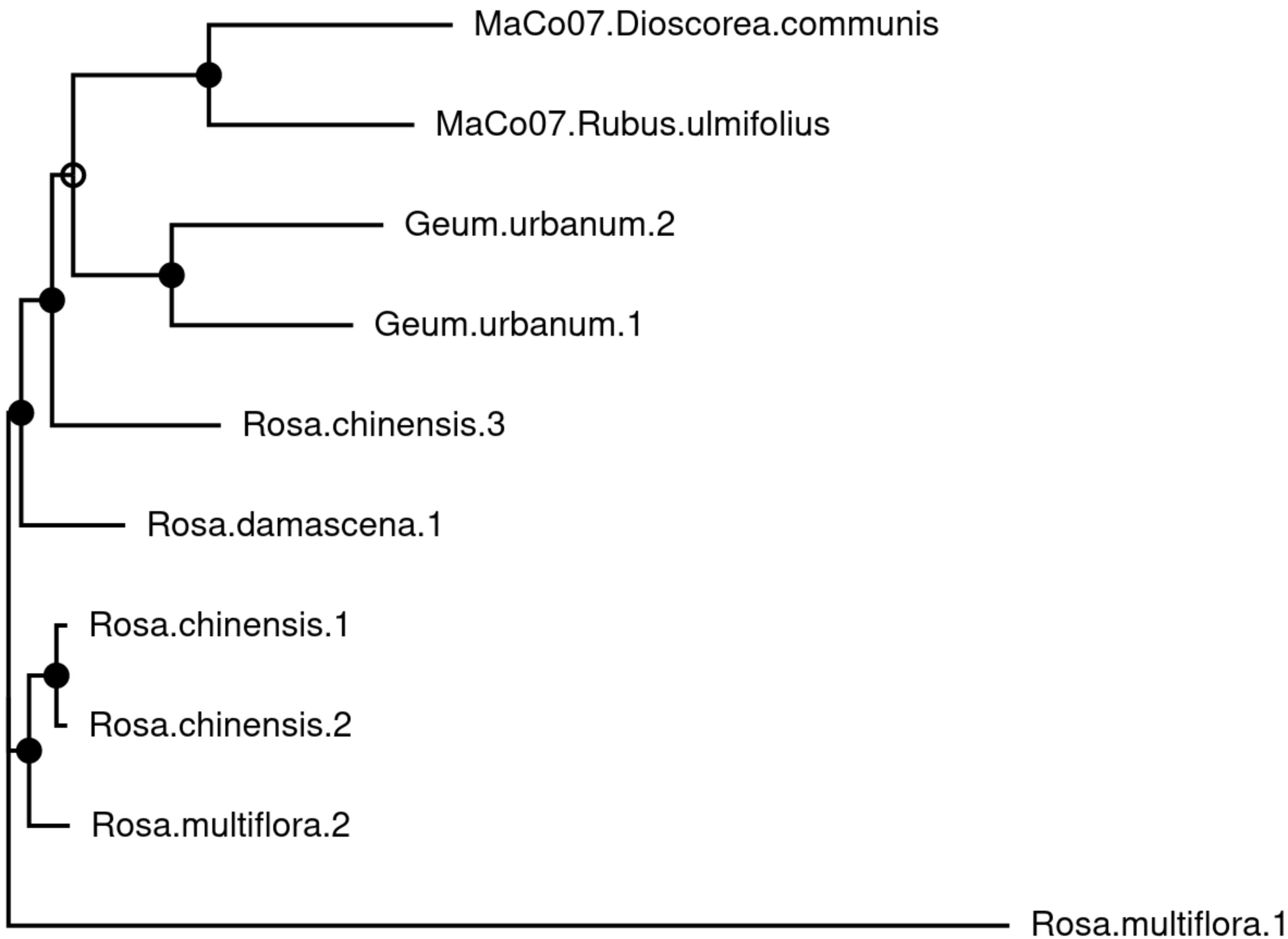

0.03

Supplement: S8 Fig — Nodes supported with boostrap values above 70% are indicated with a black dot. Nodes with bootstrap values under 70% are indicated with white dot. (PDF) [file pgen.1010964.s008.pdf]

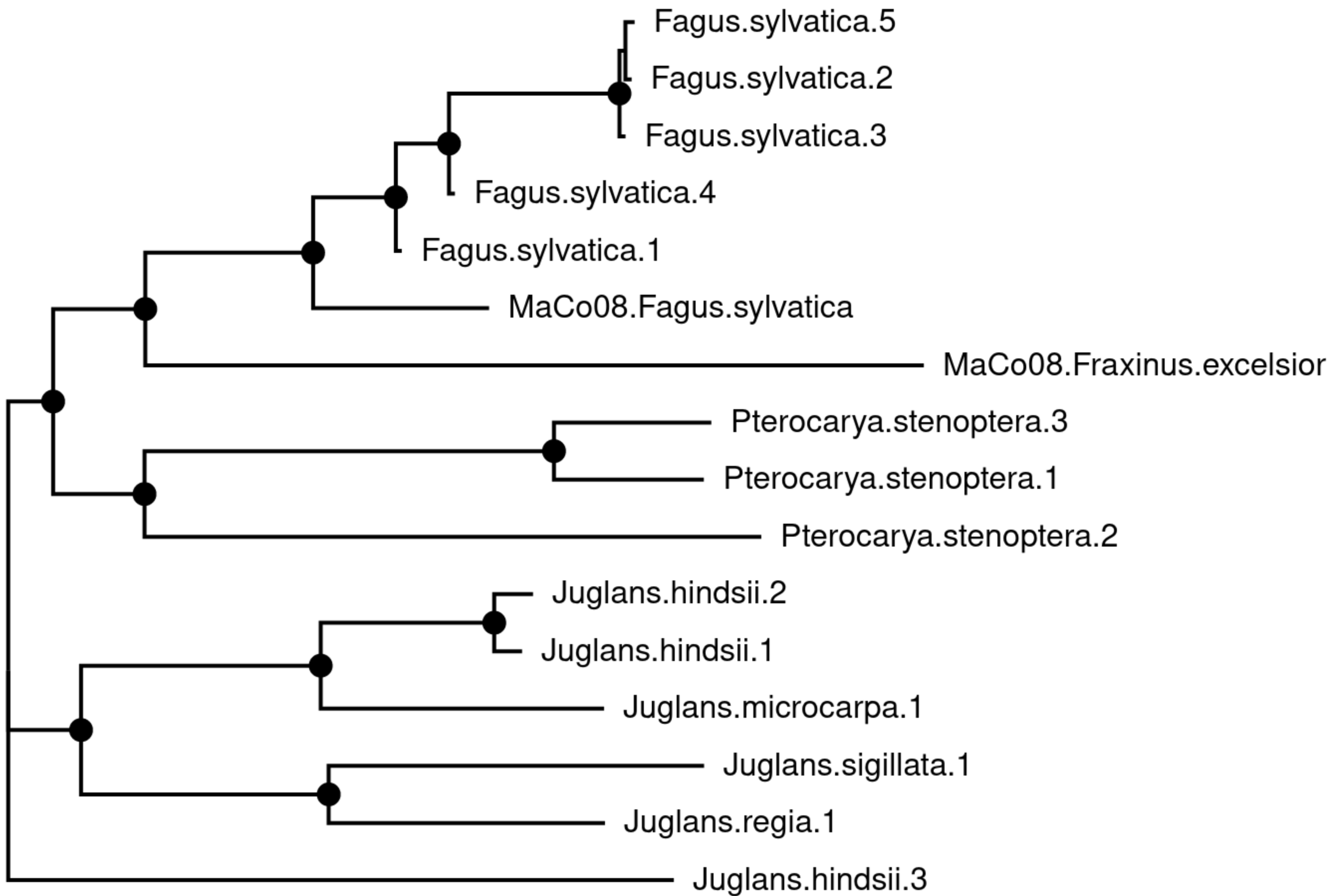

0.02

Supplement: S9 Fig — Nodes supported with boostrap values above 70% are indicated with a black dot. Nodes with bootstrap values under 70% are indicated with white dot. (PDF) [file pgen.1010964.s009.pdf]

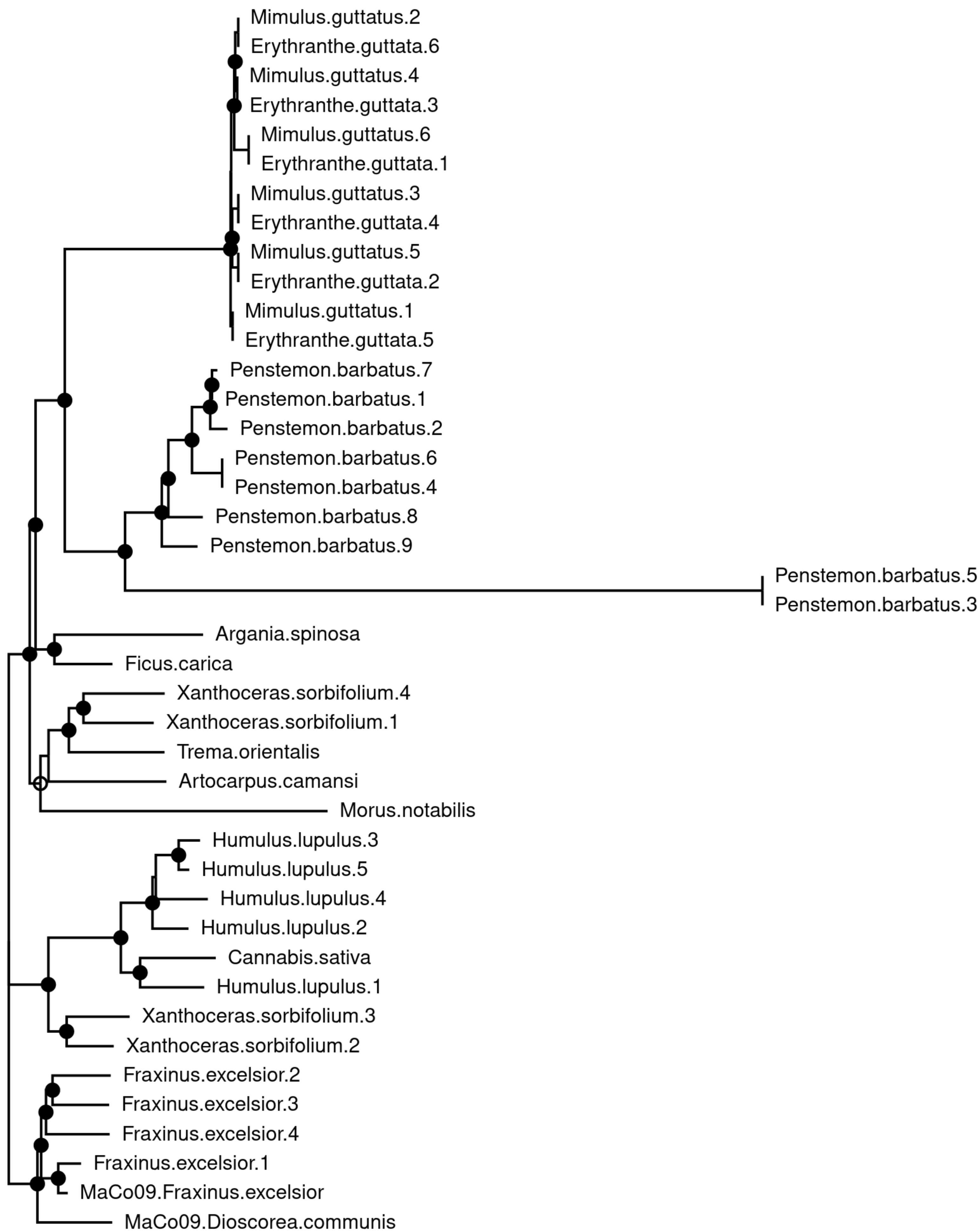

Supplement: S10 Fig — Nodes supported with boostrap values above 70% are indicated with a black dot. Nodes with bootstrap values under 70% are indicated with white dot. (PDF) [file pgen.1010964.s010.pdf]

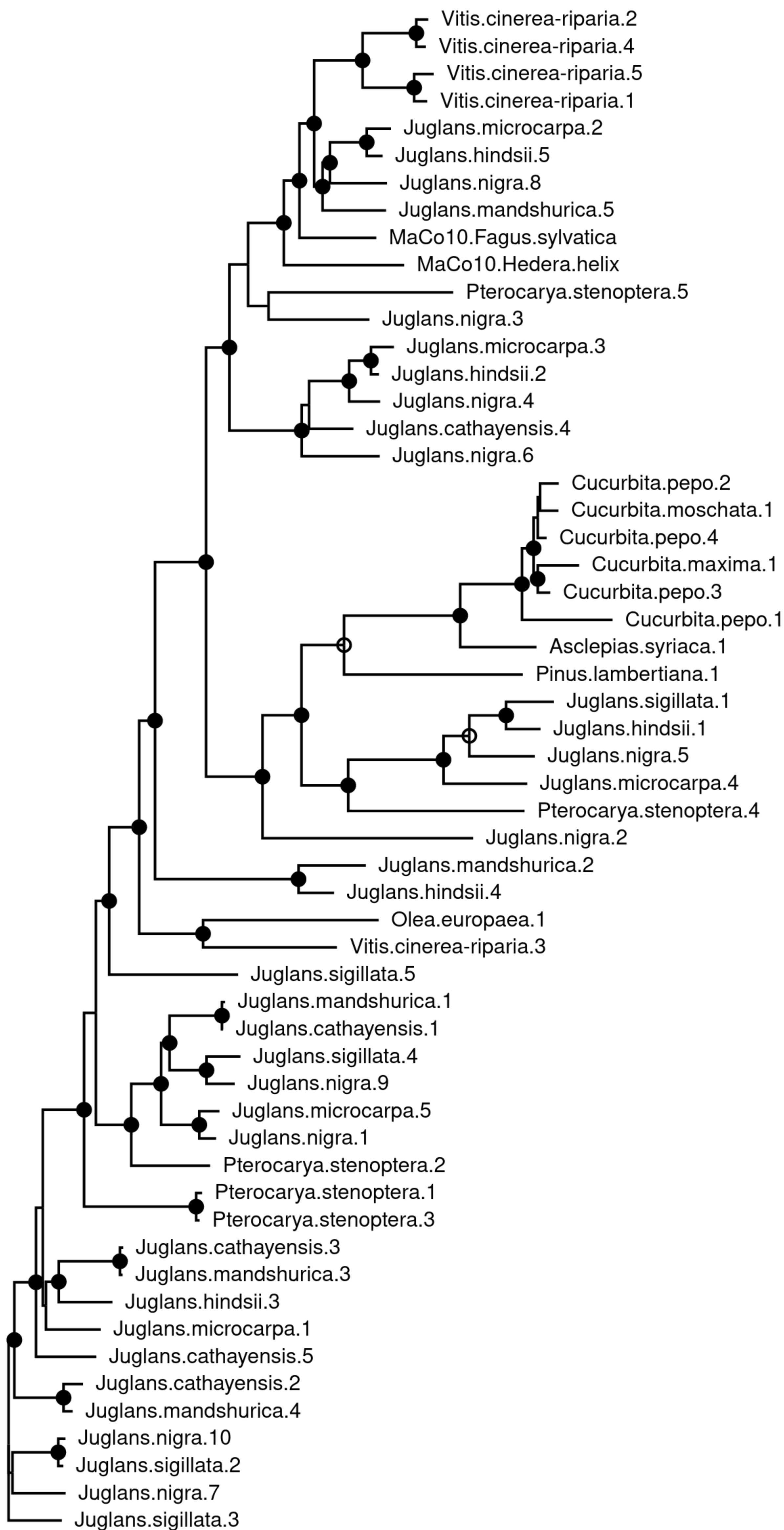

0.09

Supplement: S11 Fig — Nodes supported with boostrap values above 70% are indicated with a black dot. Nodes with bootstrap values under 70% are indicated with white dot. (PDF) [file pgen.1010964.s011.pdf]

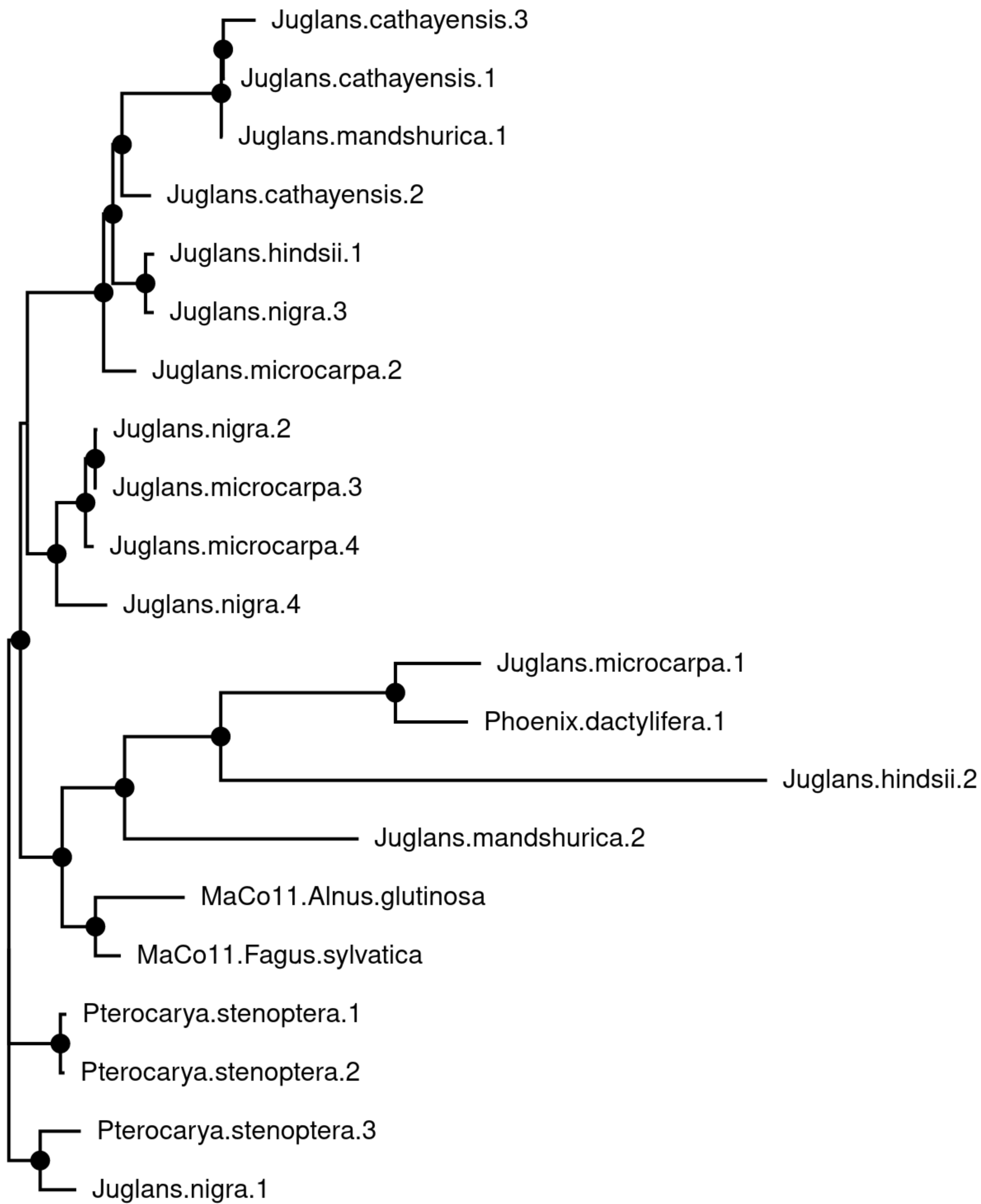

0.2

Supplement: S12 Fig — Nodes supported with boostrap values above 70% are indicated with a black dot. Nodes with bootstrap values under 70% are indicated with white dot. (PDF) [file pgen.1010964.s012.pdf]

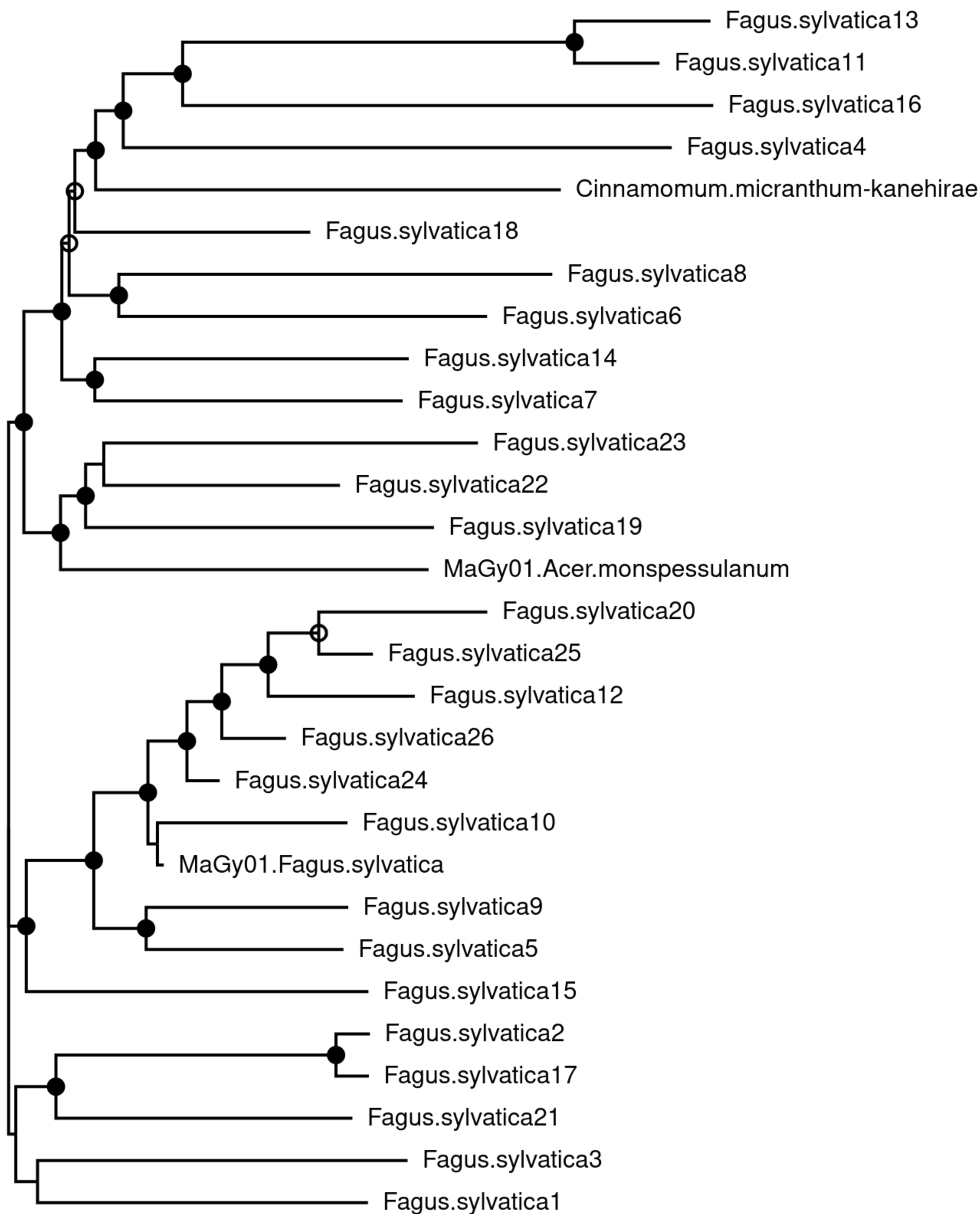

0.01

Supplement: S13 Fig — Nodes supported with boostrap values above 70% are indicated with a black dot. Nodes with bootstrap values under 70% are indicated with white dot. (PDF) [file pgen.1010964.s013.pdf]

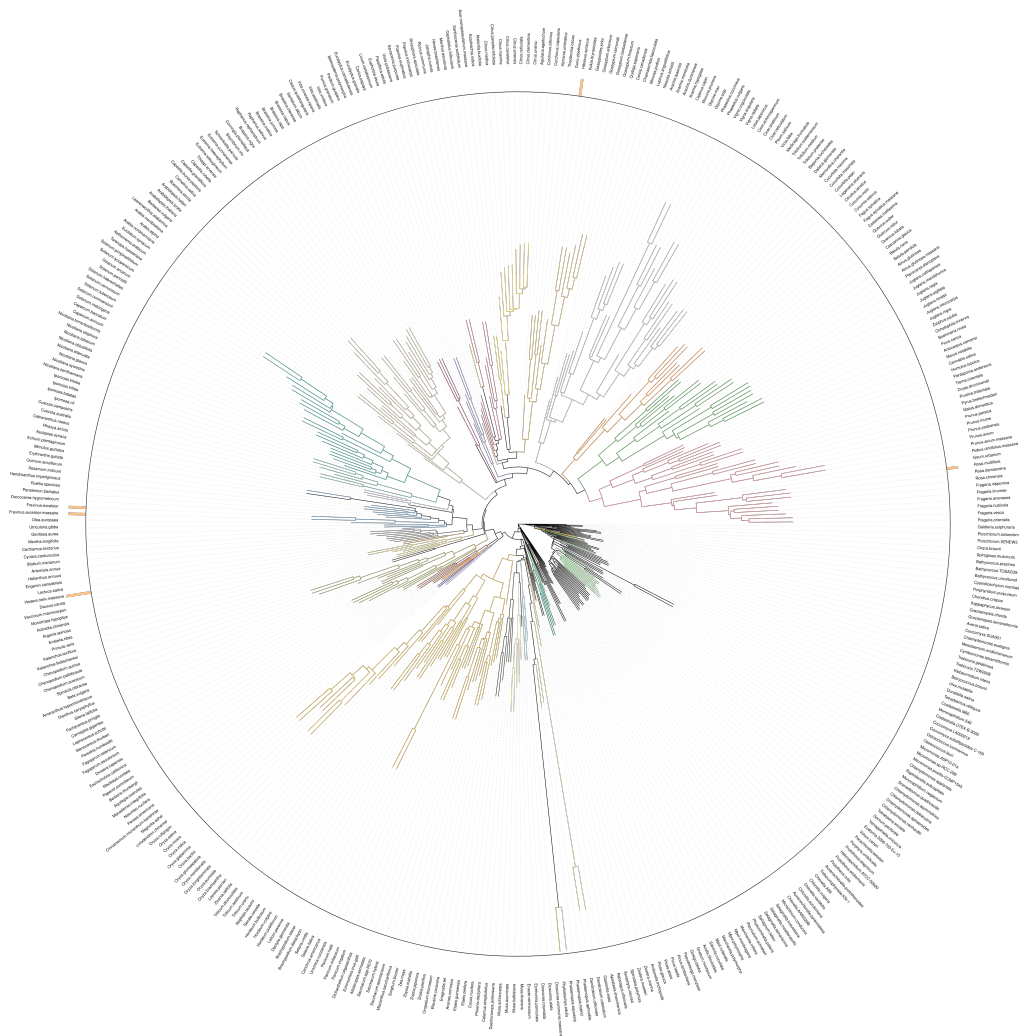

Supplement: S15 Fig — The green bars represent the species harboring the LTR family and their height the relative abundance in the host genome. (PDF) [file pgen.1010964.s015.pdf]

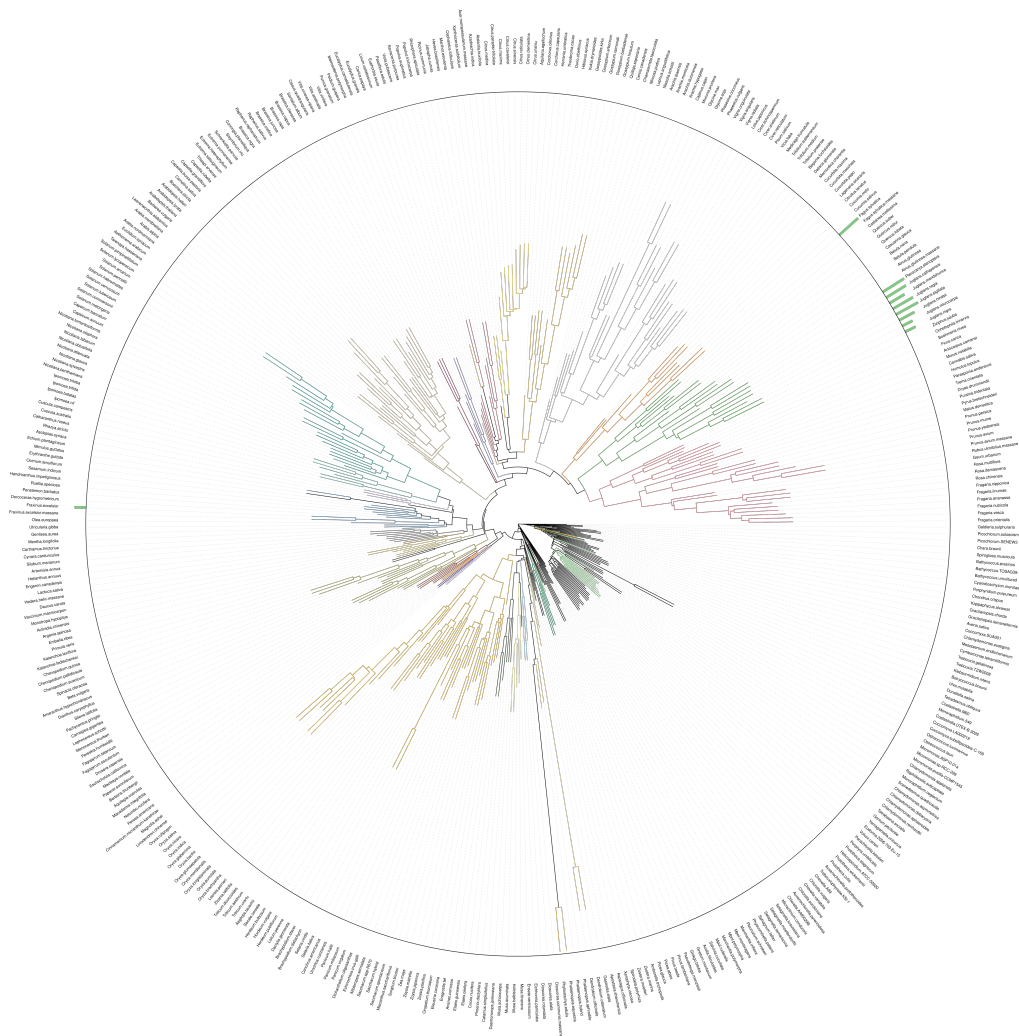

Supplement: S16 Fig — The green bars represent the species harboring the LTR family and their height the relative abundance in the host genome. (PDF) [file pgen.1010964.s016.pdf]

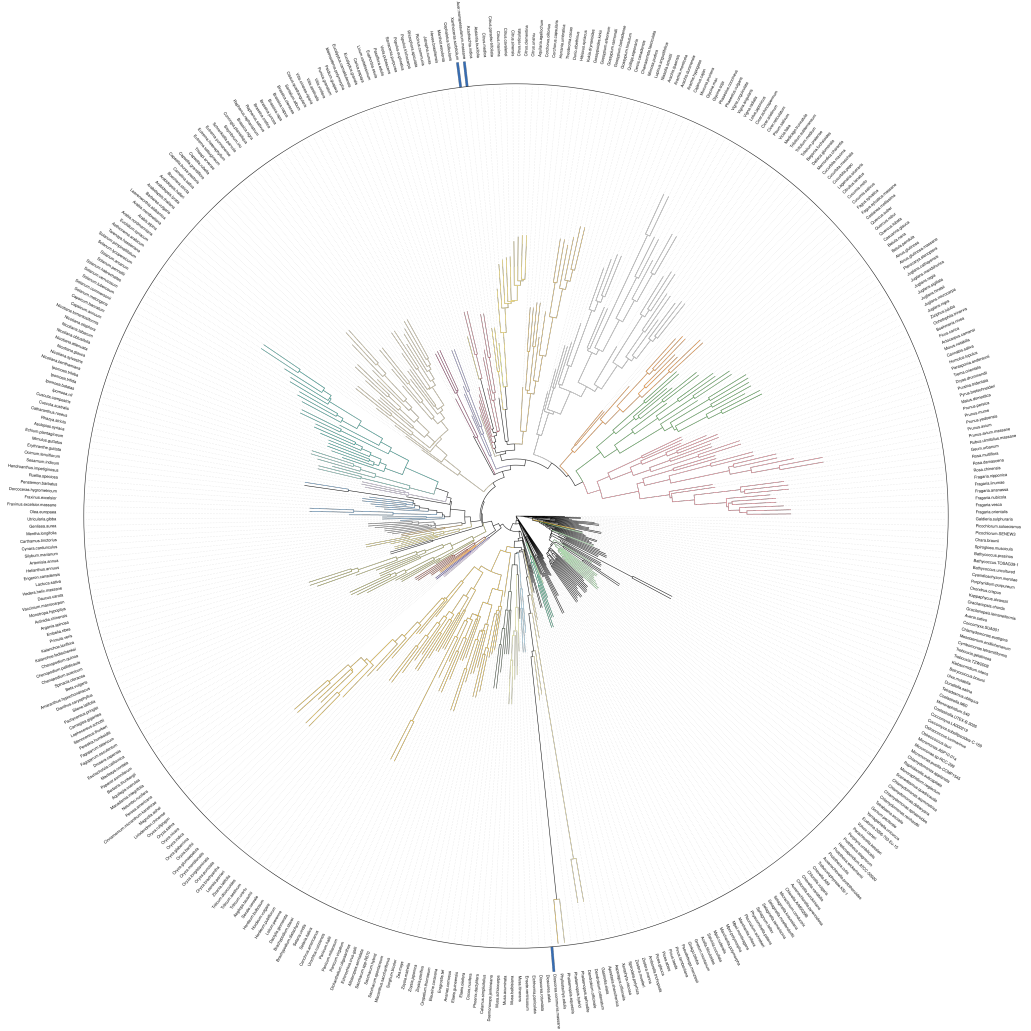

Supplement: S17 Fig — The green bars represent the species harboring the LTR family and their height the relative abundance in the host genome. (PDF) [file pgen.1010964.s017.pdf]

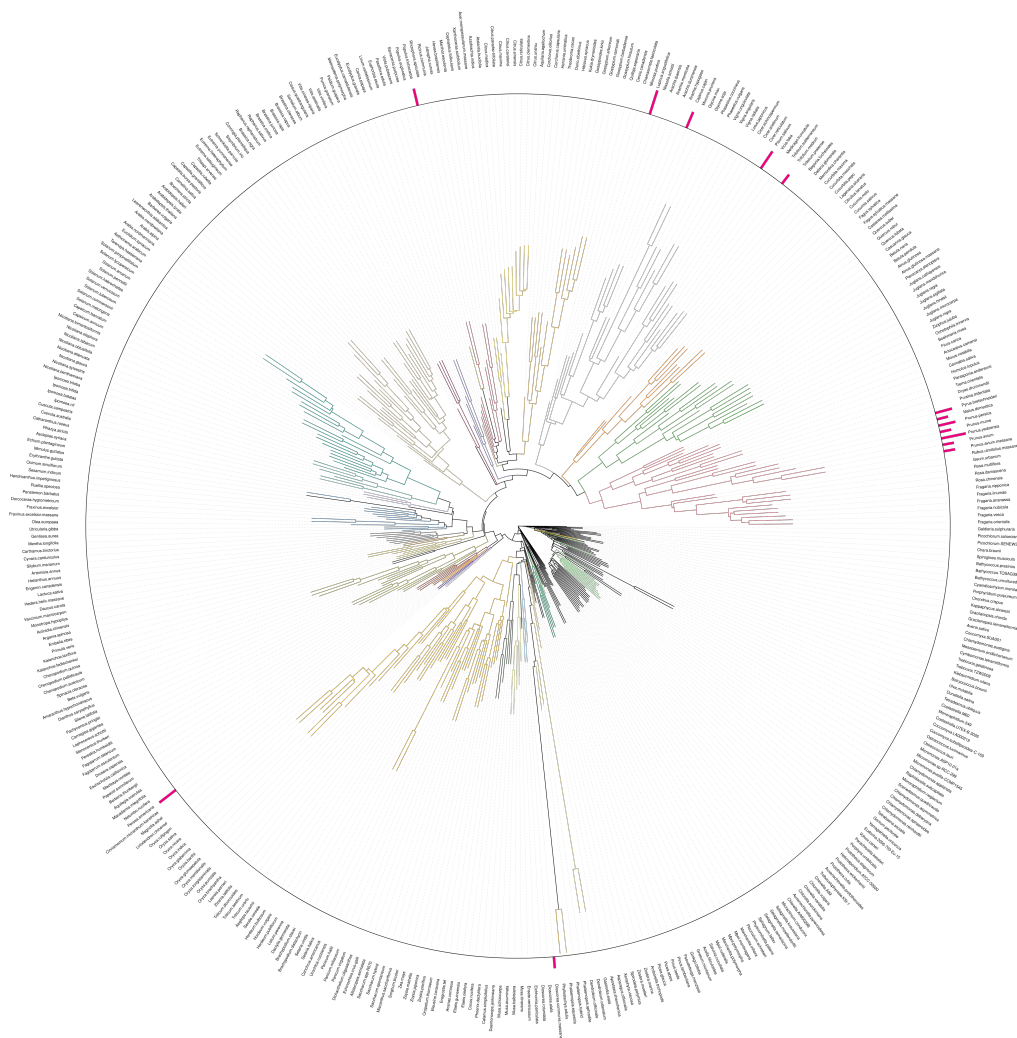

Supplement: S19 Fig — The green bars represent the species harboring the LTR family and their height the relative abundance in the host genome. (PDF) [file pgen.1010964.s019.pdf]

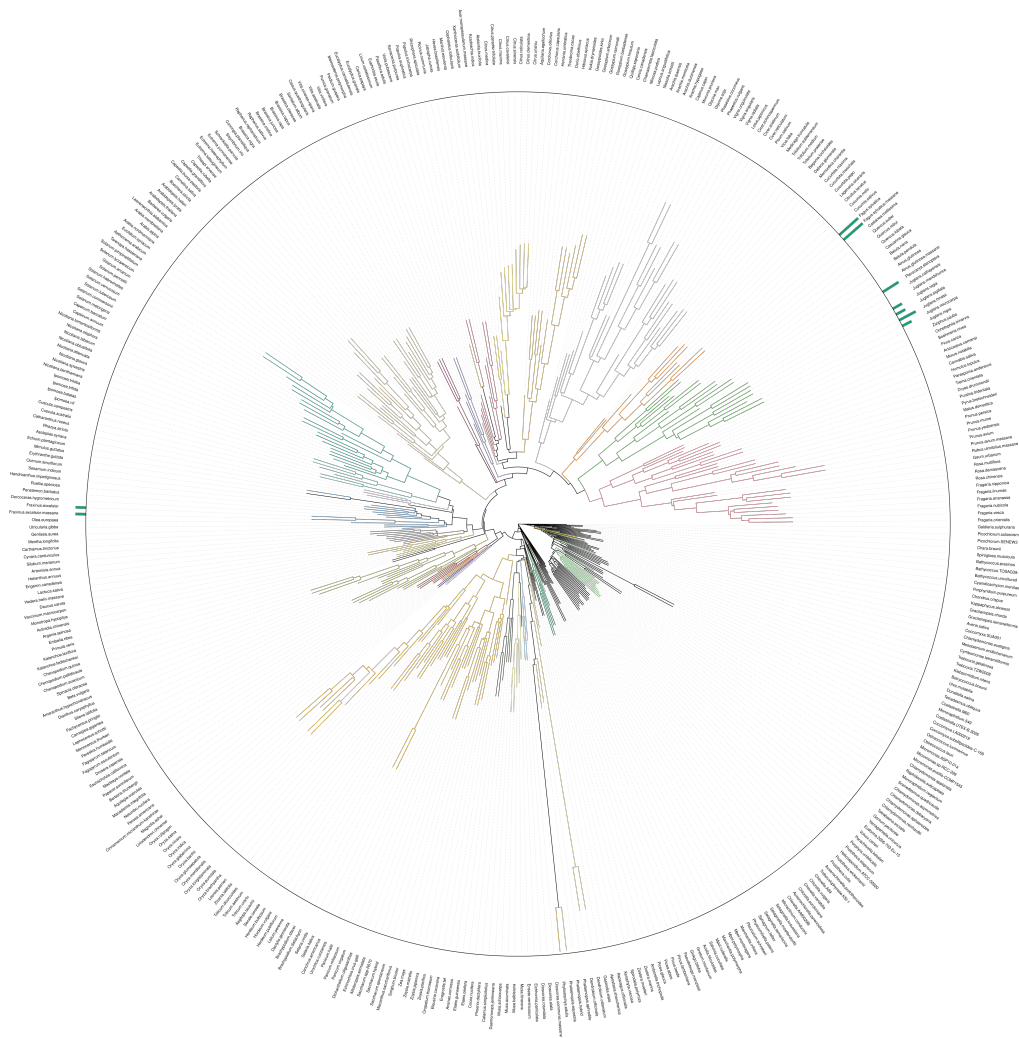

Supplement: S21 Fig — The green bars represent the species harboring the LTR family and their height the relative abundance in the host genome. (PDF) [file pgen.1010964.s021.pdf]

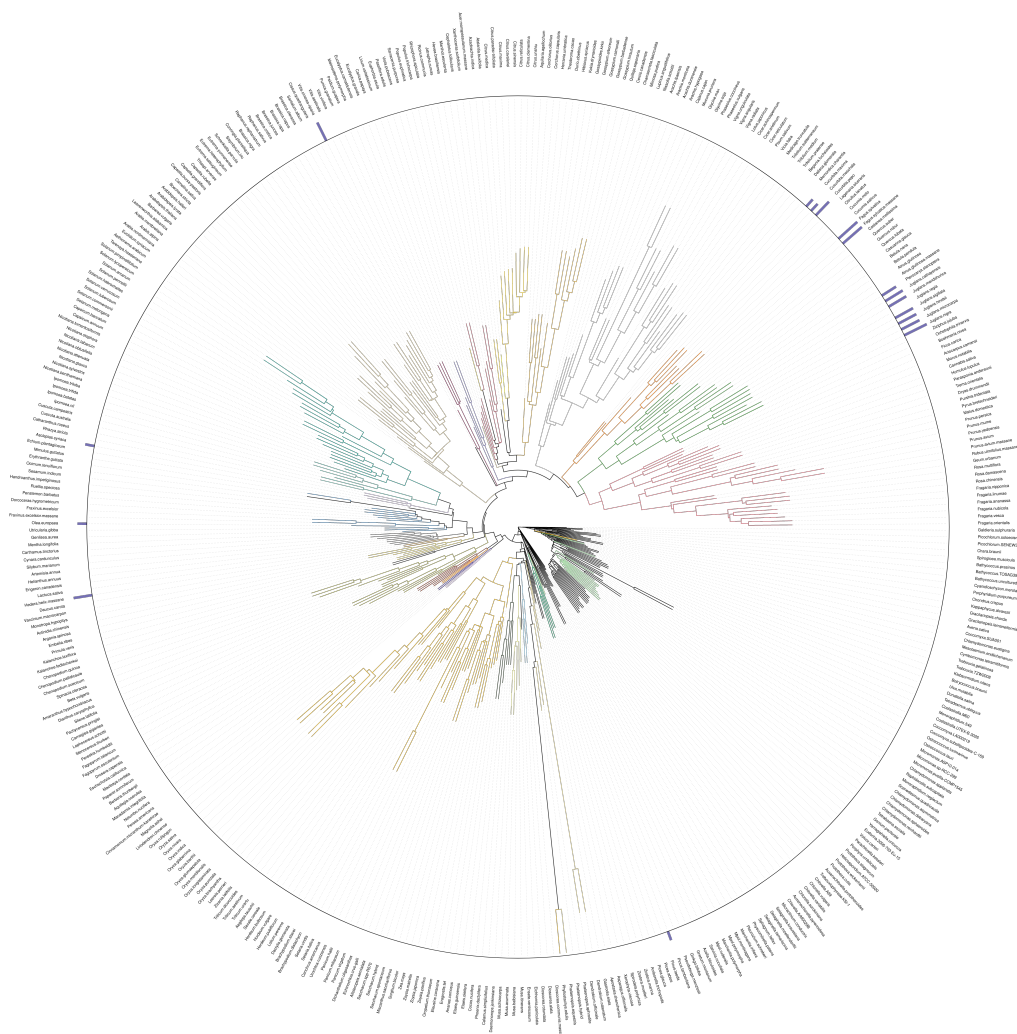

Supplement: S23 Fig — The green bars represent the species harboring the LTR family and their height the relative abundance in the host genome. (PDF) [file pgen.1010964.s023.pdf]

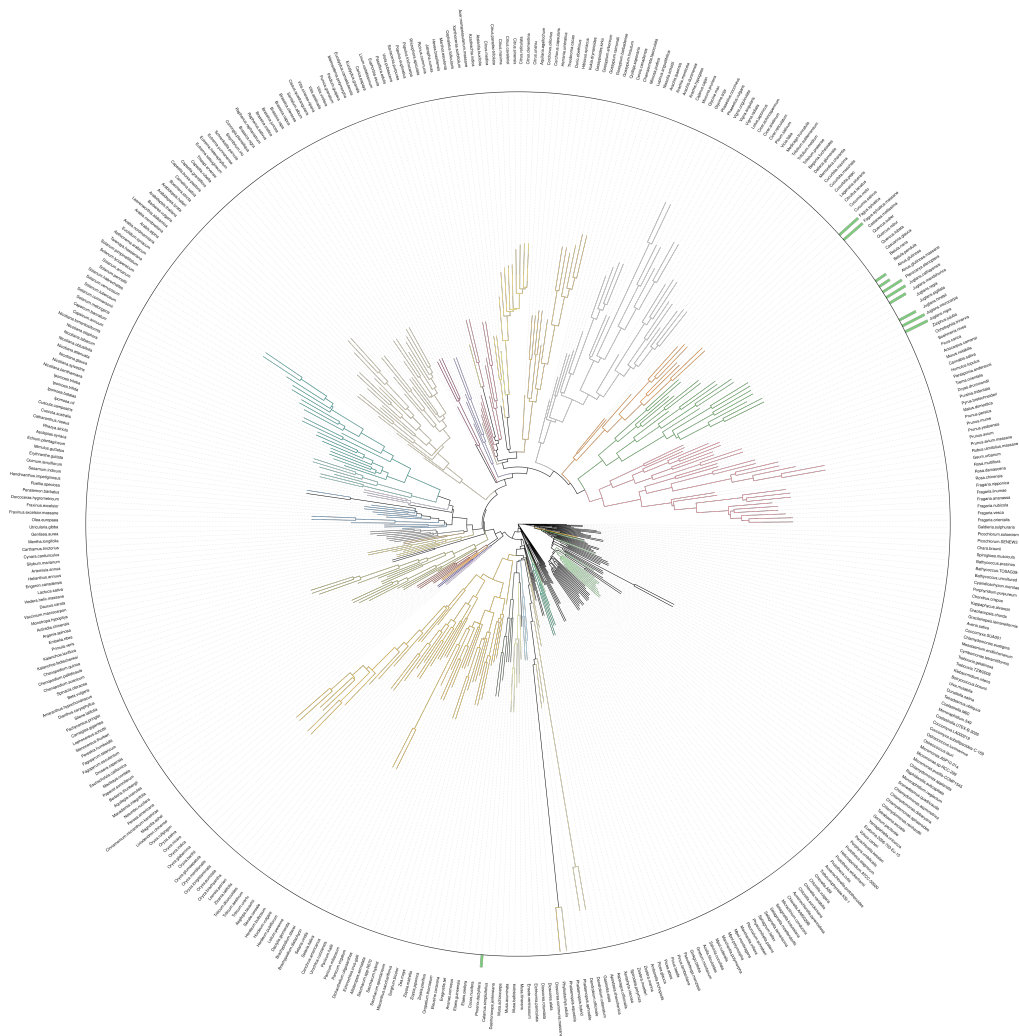

Supplement: S24 Fig — The green bars represent the species harboring the LTR family and their height the relative abundance in the host genome. (PDF) [file pgen.1010964.s024.pdf]
