# Supplementary material for: Genome-wide analysis of horizontal transfer in non-model wild species from a natural ecosystem reveals new insights into genetic exchange in plants
Source: PLoS Genet. 2023 Oct 19;19(10):e1010964. doi: 10.1371/journal.pgen.1010964 (PMC10586619; doi:10.1371/journal.pgen.1010964)
Supplement: S26 Fig — Migration direction is from top to bottom. The red arrows indicate the primers designed to amplify different regions of the transferred LTR-RTs in the species involved in the HTs. For each species, PCR was performed using DNA from two different individuals, different from those used for genome sequencing, to limit possible contamination. (PDF) [file pgen.1010964.s026.pdf]

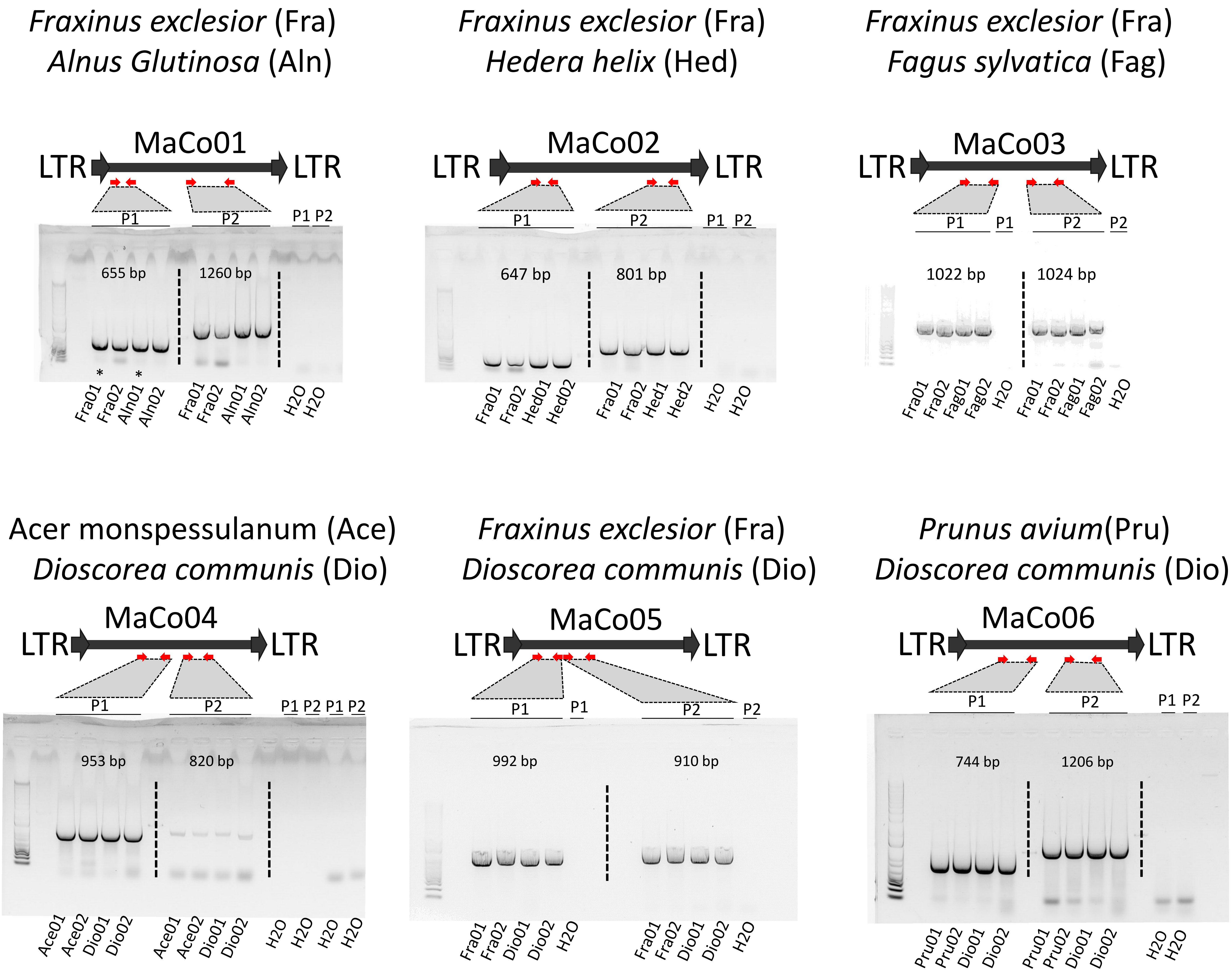
Fraxinus exclesior (Fra)
Alnus Glutinosa (Aln)
MaCo01
LTR
LTR
P1
P2
P1 P2
655 bp
1260 bp
*
*
Fra01 Fra02 Aln01 Aln02 Fra01 Fra02 Aln01 Aln02 H2O H2O
Fraxinus exclesior (Fra)
Hedera helix (Hed)
MaCo02
LTR
LTR
P1
P2
P1 P2
647 bp
801 bp
Fra01 Fra02 Hed01 Hed02 Fra01 Fra02 Hed1 Hed2 H2O H2O
Fraxinus exclesior (Fra)
Fagus sylvatica (Fag)
MaCo03
LTR
LTR
P1
P1
P2
P2
1022 bp
1024 bp
Fra01 Fra02 Fag01 Fag02 H2O Fra01 Fra02 Fag01 Fag02 H2O
Acer monspessulanum (Ace)
Dioscorea communis (Dio)
MaCo04
LTR
LTR
P1
P2
P1 P2 P1 P2
953 bp
820 bp
Ace01 Ace02 Dio01 Dio02 Ace01 Ace02 Dio01 Dio02 H2O H2O H2O H2O
Fraxinus exclesior (Fra)
Dioscorea communis (Dio)
MaCo05
LTR
LTR
P1
P1
P2
P2
992 bp
910 bp
Fra01 Fra02 Dio01 Dio02 H2O Fra01 Fra02 Dio01 Dio02 H2O
Prunus avium(Pru)
Dioscorea communis (Dio)
MaCo06
LTR
LTR
P1
P2
P1 P2
744 bp
1206 bp
Pru01 Pru02 Dio01 Dio02 Pru01 Pru02 Dio01 Dio02 H2O H2O
