# Supplementary material for: Genome-wide analysis of horizontal transfer in non-model wild species from a natural ecosystem reveals new insights into genetic exchange in plants
Source: PLoS Genet. 2023 Oct 19;19(10):e1010964. doi: 10.1371/journal.pgen.1010964 (PMC10586619; doi:10.1371/journal.pgen.1010964)
Supplement: S27 Fig — Migration direction is from top to bottom. The red arrows indicate the primers designed to amplify different regions of the transferred LTR-RTs in the species involved in the HTs. For each species, PCR was performed using DNA from two different individuals, different from those used for genome sequencing, to limit possible contamination. (PDF) [file pgen.1010964.s027.pdf]

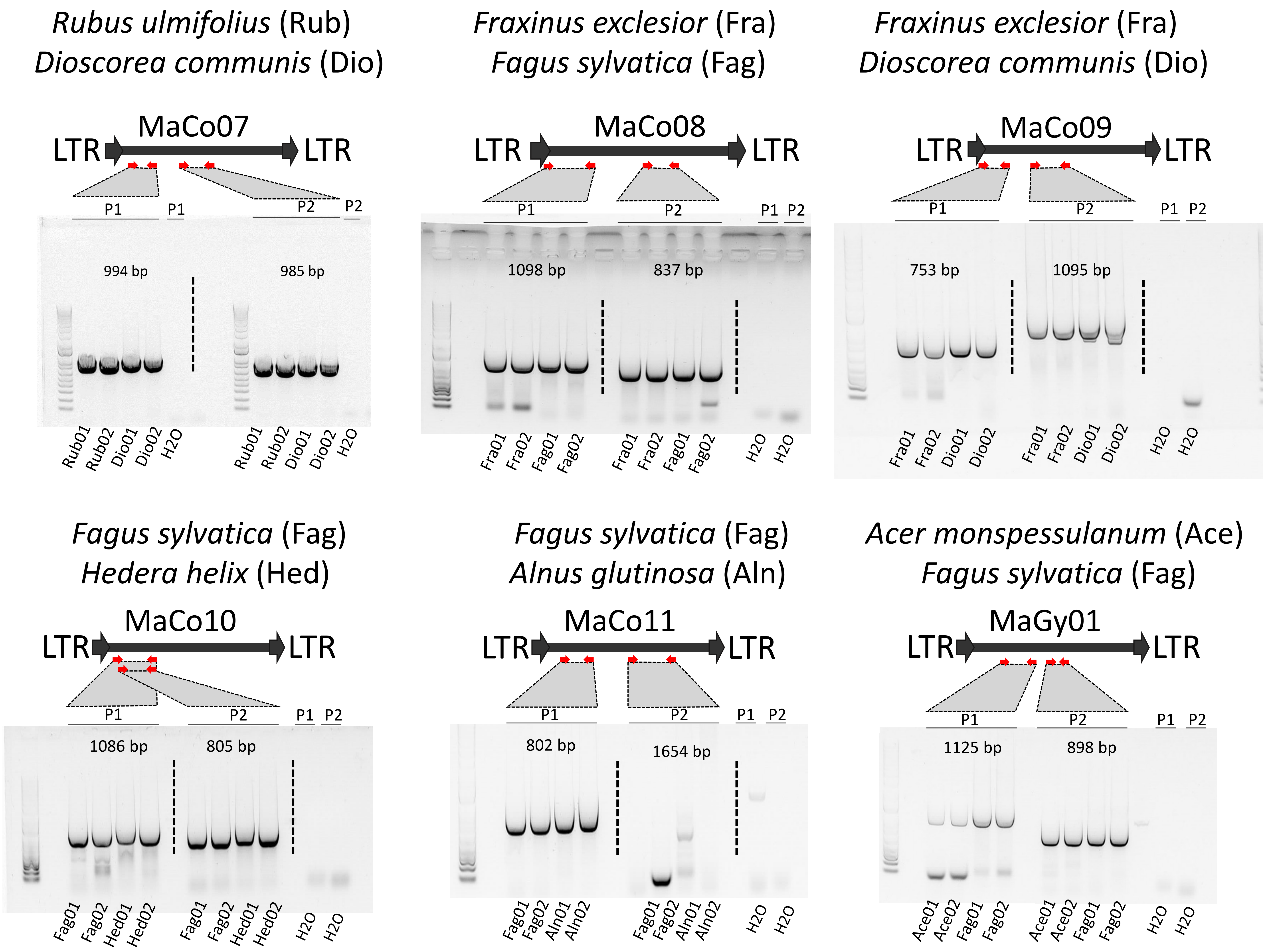
Rubus ulmifolius (Rub)
Dioscorea communis (Dio)
MaCo07
LTR
LTR
P1
P1
P2
P2
994 bp
985 bp
Rub01 Rub02 Dio01 Dio02 H2O
Rub01 Rub02 Dio01 Dio02 H2O
Fraxinus exclesior (Fra)
Fagus sylvatica (Fag)
MaCo08
LTR
LTR
P1
P2
P1 P2
1098 bp
837 bp
Fra01 Fra02 Fag01 Fag02
Fra01 Fra02 Fag01 Fag02
H2O H2O
Fraxinus exclesior (Fra)
Dioscorea communis (Dio)
MaCo09
LTR
LTR
P1
P2
P1 P2
753 bp
1095 bp
Fra01 Fra02 Dio01 Dio02
Fra01 Fra02 Dio01 Dio02
H2O H2O
Fagus sylvatica (Fag)
Hedera helix (Hed)
MaCo10
LTR
LTR
P1
P2
P1 P2
1086 bp
805 bp
Fag01 Fag02 Hed01 Hed02
Fag01 Fag02 Hed01 Hed02
H2O H2O
Fagus sylvatica (Fag)
Alnus glutinosa (Aln)
MaCo11
LTR
LTR
P1
P2
P1 P2
802 bp
1654 bp
Fag01 Fag02 Aln01 Aln02
Fag01 Fag02 Aln01 Aln02
H2O H2O
Acer monspessulanum (Ace)
Fagus sylvatica (Fag)
MaGy01
LTR
LTR
P1
P2
P1 P2
1125 bp
898 bp
Ace01 Ace02 Fag01 Fag02
Ace01 Ace02 Fag01 Fag02
H2O H2O
