# Supplementary material for: Genome-wide analysis of horizontal transfer in non-model wild species from a natural ecosystem reveals new insights into genetic exchange in plants
Source: PLoS Genet. 2023 Oct 19;19(10):e1010964. doi: 10.1371/journal.pgen.1010964 (PMC10586619; doi:10.1371/journal.pgen.1010964)
Supplement: S28 Fig — Visual representation was achieved using http://kablammo.wasmuthlab.org/ software. License: https://github.com/jwintersinger/kablammo/blob/master/LICENSE (PDF) [file pgen.1010964.s028.pdf]

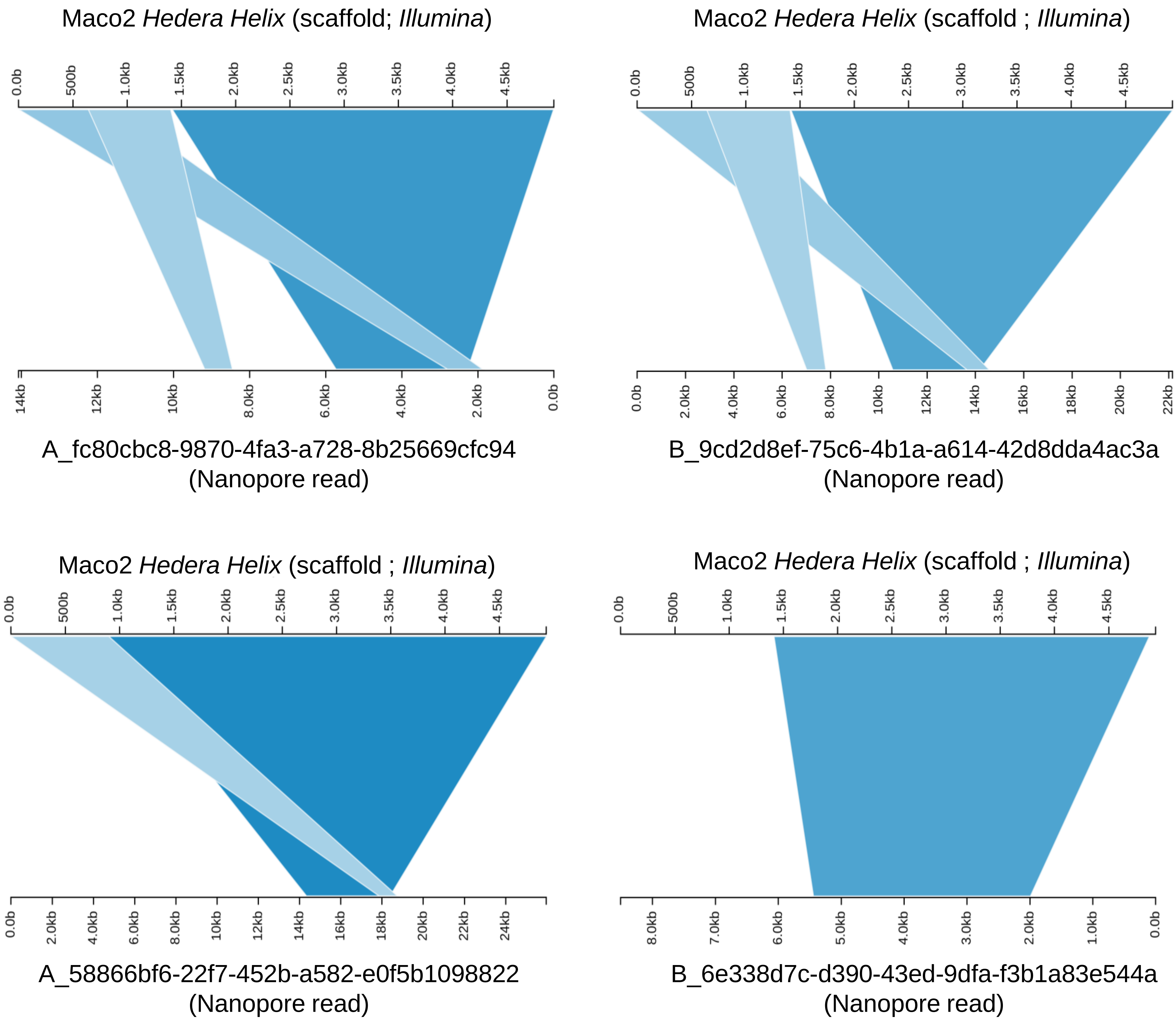
Maco2 Hedera Helix (scaffold; Illumina)
0.0b
500b
1.0kb
1.5kb
2.0kb
2.5kb
3.0kb
3.5kb
4.0kb
4.5kb
14kb
12kb
10kb
8.0kb
6.0kb
4.0kb
2.0kb
0.0b
A_fc80cbc8-9870-4fa3-a728-8b25669cfc94
(Nanopore read)
Maco2 Hedera Helix (scaffold ; Illumina)
0.0b
500b
1.0kb
1.5kb
2.0kb
2.5kb
3.0kb
3.5kb
4.0kb
4.5kb
0.0b
2.0kb
4.0kb
6.0kb
8.0kb
10kb
12kb
14kb
16kb
18kb
20kb
22kb
B_9cd2d8ef-75c6-4b1a-a614-42d8dda4ac3a
(Nanopore read)
Maco2 Hedera Helix (scaffold ; Illumina)
0.0b
500b
1.0kb
1.5kb
2.0kb
2.5kb
3.0kb
3.5kb
4.0kb
4.5kb
0.0b
2.0kb
4.0kb
6.0kb
8.0kb
10kb
12kb
14kb
16kb
18kb
20kb
22kb
24kb
A_58866bf6-22f7-452b-a582-e0f5b1098822
(Nanopore read)
Maco2 Hedera Helix (scaffold ; Illumina)
0.0b
500b
1.0kb
1.5kb
2.0kb
2.5kb
3.0kb
3.5kb
4.0kb
4.5kb
8.0kb
7.0kb
6.0kb
5.0kb
4.0kb
3.0kb
2.0kb
1.0kb
0.0b
B_6e338d7c-d390-43ed-9dfa-f3b1a83e544a
(Nanopore read)
