# Supplementary material for: Genome-wide analysis of horizontal transfer in non-model wild species from a natural ecosystem reveals new insights into genetic exchange in plants
Source: PLoS Genet. 2023 Oct 19;19(10):e1010964. doi: 10.1371/journal.pgen.1010964 (PMC10586619; doi:10.1371/journal.pgen.1010964)
Supplement: S29 Fig — Visual representation was achieved using http://kablammo.wasmuthlab.org/ software. License: https://github.com/jwintersinger/kablammo/blob/master/LICENSE. (PDF) [file pgen.1010964.s029.pdf]

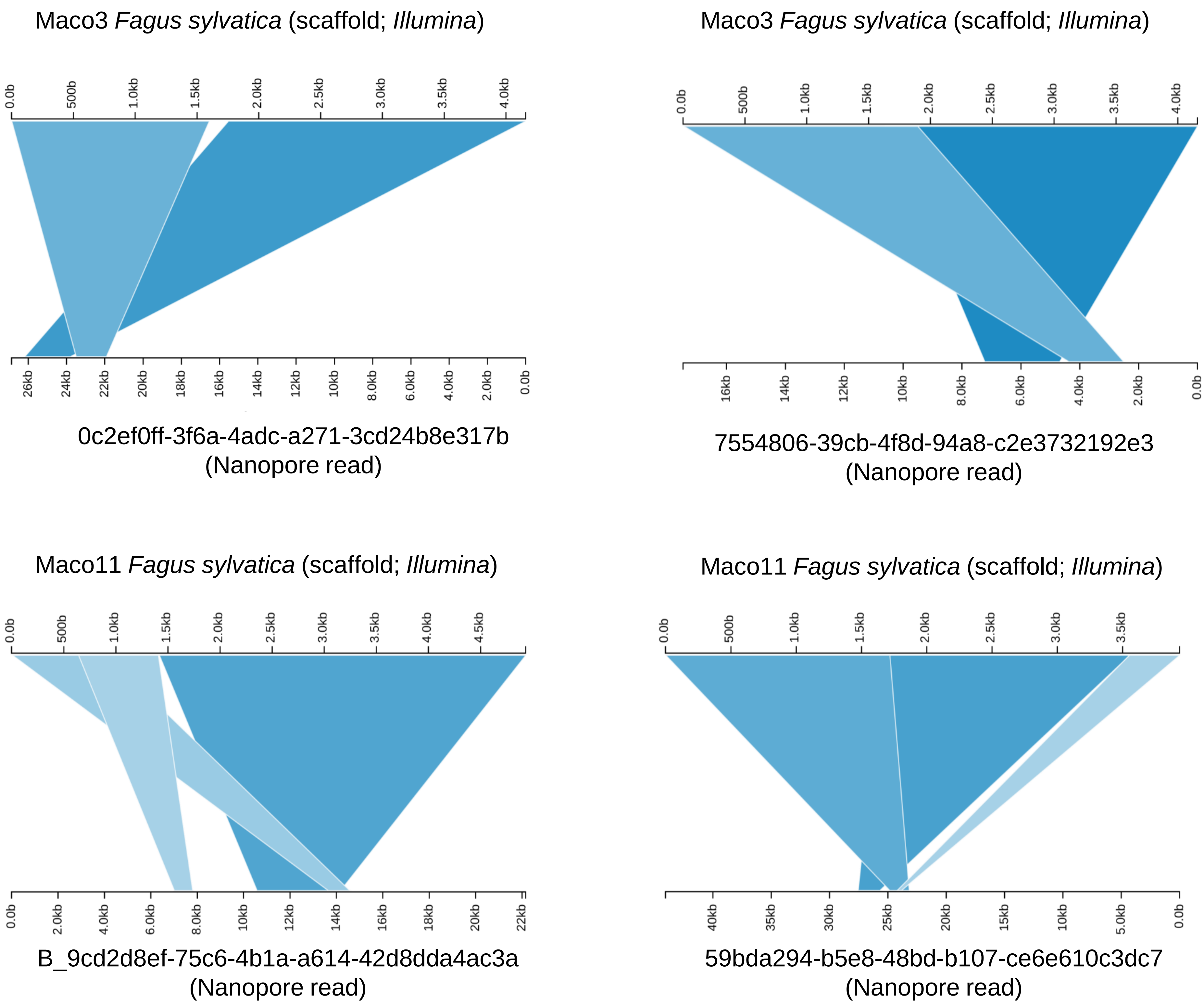
Maco3 *Fagus sylvatica* (scaffold; *Illumina*)
0.0b 500b 1.0kb 1.5kb 2.0kb 2.5kb 3.0kb 3.5kb 4.0kb
26kb 24kb 22kb 20kb 18kb 16kb 14kb 12kb 10kb 8.0kb 6.0kb 4.0kb 2.0kb 0.0b
0c2ef0ff-3f6a-4adc-a271-3cd24b8e317b
(Nanopore read)
Maco3 *Fagus sylvatica* (scaffold; *Illumina*)
0.0b 500b 1.0kb 1.5kb 2.0kb 2.5kb 3.0kb 3.5kb 4.0kb
16kb 14kb 12kb 10kb 8.0kb 6.0kb 4.0kb 2.0kb 0.0b
7554806-39cb-4f8d-94a8-c2e3732192e3
(Nanopore read)
Maco11 *Fagus sylvatica* (scaffold; *Illumina*)
0.0b 500b 1.0kb 1.5kb 2.0kb 2.5kb 3.0kb 3.5kb 4.0kb 4.5kb
0.0b 2.0kb 4.0kb 6.0kb 8.0kb 10kb 12kb 14kb 16kb 18kb 20kb 22kb
B_9cd2d8ef-75c6-4b1a-a614-42d8dda4ac3a
(Nanopore read)
Maco11 *Fagus sylvatica* (scaffold; *Illumina*)
0.0b 500b 1.0kb 1.5kb 2.0kb 2.5kb 3.0kb 3.5kb
40kb 35kb 30kb 25kb 20kb 15kb 10kb 5.0kb 0.0b
59bda294-b5e8-48bd-b107-ce6e610c3dc7
(Nanopore read)
