# Supplementary material for: Genome-wide analysis of horizontal transfer in non-model wild species from a natural ecosystem reveals new insights into genetic exchange in plants
Source: PLoS Genet. 2023 Oct 19;19(10):e1010964. doi: 10.1371/journal.pgen.1010964 (PMC10586619; doi:10.1371/journal.pgen.1010964)

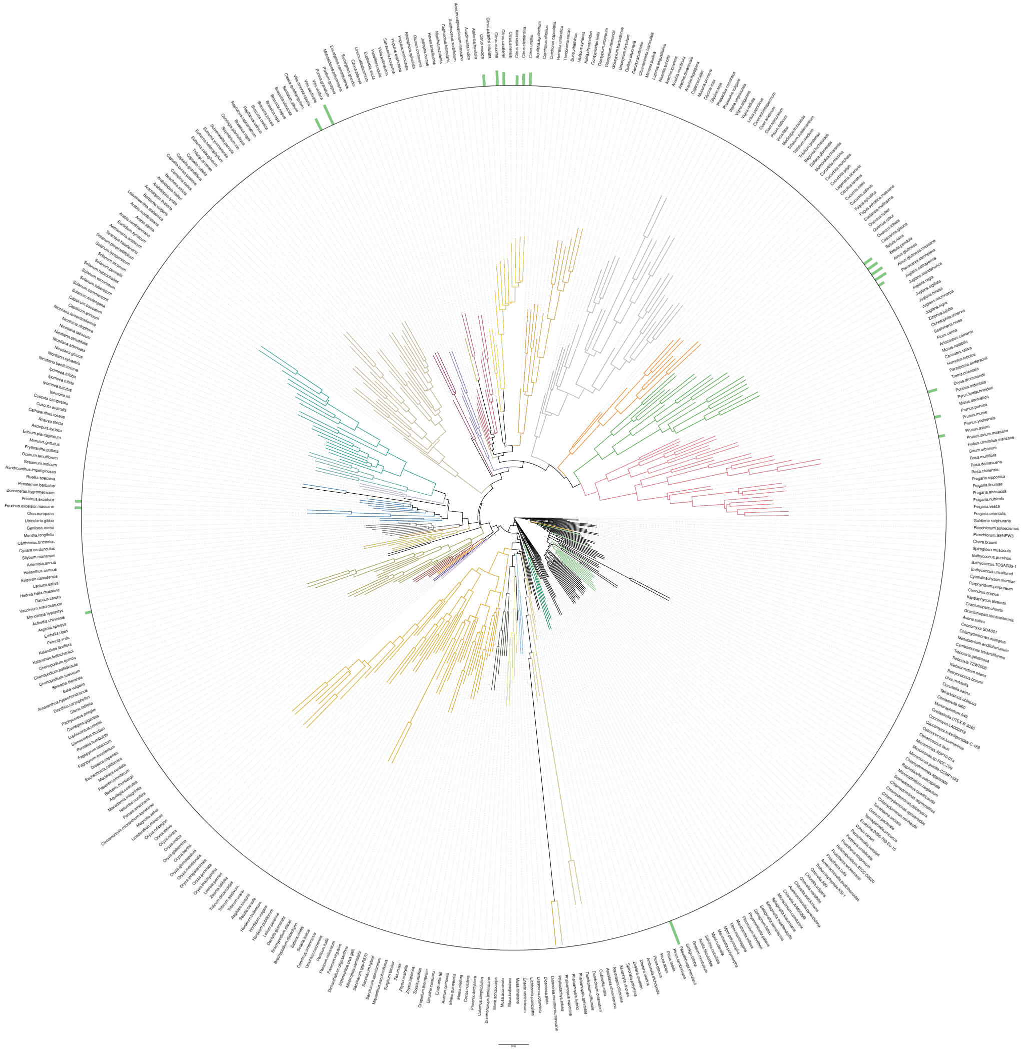

Supplement: S14 Fig — The green bars represent the species harboring the LTR family and their height the relative abundance in the host genome. (PDF) [file pgen.1010964.s014.pdf]
