# Supplementary material for: Genome-wide analysis of horizontal transfer in non-model wild species from a natural ecosystem reveals new insights into genetic exchange in plants
Source: PLoS Genet. 2023 Oct 19;19(10):e1010964. doi: 10.1371/journal.pgen.1010964 (PMC10586619; doi:10.1371/journal.pgen.1010964)
Supplement: Attachment — Submitted filename: Response.to.reviewers.docx [file pgen.1010964.s042.docx]

**Reviewer #1:**

« This is a description of a pilot study designed to test the hypothesis that relatively inexpensive Illumina sequencing can be rapidly detect horizontal transfer events from non-model species. This would facilitate more wide-spread detection of horizontal transfer in natural settings. In theory, surveys of those populations performed for other reasons could be subject to this analysis pipeline. The authors have convincingly demonstrated that they can in fact use this pipeline for that purpose. »

Response : We thank you for your understanding and appreciation of the objectives and results of our work.

« My concern here is that although this will be of interest for a specialized audience, the results themselves are not significantly different from those already obtained in previous studies. »

Response : We acknowledge that at a first glance our present study might seem to be not significantly different from findings presented in our previous Genome Research paper (El baidouri et al 2014) and other earlier studies. However, we would like to emphasize that this is not the case for several reasons:

1- Our study is the first to provide clear evidence that LTR-retrotransposons are the primary genetic elements horizontally transferred between plant species. We were able to assert this claim through the application of an innovative pipeline. This tool empowers us to detect HTs on a whole-genome scale without introducing any biases in favor of TEs or Gene.

2- We have utilized a unique approach designed specifically for identification of HTs directly from Illumina's raw genome-wide data. This sets our study apart from previous studies that relied on traditional methods.

3- In addition to these innovative techniques, we have made another important discovery. Contrary to previous beliefs, not all LTR-retrotransposon families are capable of HT. Our results have shown that mainly two *Copia* lineages—Ale and Ivana—have the capability for horizontal transfer between plants. This finding presents a significant advance in our understanding of HT specificities among non-parasitic plants.

4- Last but not least our study is the first to provide substantial evidence demonstrating that lianas have been subject to numerous and frequent horizontal transfer events with tree species. This uncovers a new route for HT among non-parasitic plants, thereby expanding our comprehension of of the nature of biotic contact promoting HT other than parasitism.

In light of these distinct and important findings, we firmly believe that our study provides novel insights and significant additions to the existing body of knowledge on the subject.

I could have imagined that the same protocol could be used in environment more likely to be illuminating, such as an island environment that has undergone a series of invasions of non-native species from widely divergent places.

Response : Thank you for pointing this out. We agree that using this protocol in an island environment with native and non-native invasive species could be of great interest and may enable us to explore whether non-native species may have acquired new genetic material from their new environment. Detecting such events (recent HTs) requires moving to population-level scale, which we are currently exploring as part of an other ongoing project.

Introduction: The authors have provided numerous references supporting the idea that horizontal transfer is common; collectively, the data are quite convincing. However, I would suggest a note of caution. The evidence for HT varies widely depending on the particular study, and the caveats that were once carefully included have been abandoned. Particularly because this is a self-described pilot study, I would have expected a more thorough discussion of potential issues with HT claims upfront, rather than buried in the text (although I do appreciate that the authors do in fact address most potential issues there).

Response : We acknowledge the challenging nature of inferring horizontal transfers (HTs), as often the alternative hypotheses cannot be rejected with total confidence. Many studies predominantly rely on phylogenetic incongruence as their basis to infer HTs. However, this approach is not without its drawbacks, as it often comes with several technical and biological biases such as lineage sorting, duplications followed by gain or loss events, among others. To overcome these limitations, our study incorporates two additional criteria, as outlined in our methodology: high similarity and patchy distribution. By considering these three criteria collectively, we present compelling evidence supporting the HT hypothesis. Additionally, the identified transferred LTRs were in most cases as described in the current study more conserved than houskeeping genes. This fact is striking when considering that these repetitive elements typically do not endure the same selection pressures that genes do. The pronounced conservation of these elements is therefore intriguing and challenges traditional explanations of vertical transmission, especially when considered in the context of the extremely divergent species examined in our study. Finally, we conducted wet lab experiments to validate the identified HTs. For this purpose, we performed PCR and sequencing on multiple individuals and utilized multiple PCR primers couple to address any concerns related to contamination. This rigorous experimental approach further strengthens the support for all the HTs described in our study.

Page 3, line 28. This would be a good time to discuss other possible reasons to reject the HT hypothesis. For instance, high birth death genes, such as R genes and transposable elements (TEs) are prone to exhibit phylogenetic incongruity and patchy distribution.

Response : Thank you for your comment. We have incorporated an additional paragraph to examine potential alternative hypotheses that could challenge the horizontal transfers hypothesis (see page 4, line 2 to 11).

Page 4, line 19. This is a sensible approach, but why not also use the approach on other, well documented examples of HT? This would be of interest because it would both provide a test of the protocol and, perhaps shed additional light on those HT events. And I see that the authors did just that! However, I would note that although LTR TEs are much more likely to be subject to HT, due to their repetitive nature, high rates of birth-death, and unusual pattern of selection they may not have been the best example. I would think a functional gene that has been unambiguously demonstrated to have been horizontally transferred would have been a better test.

Response : In accordance with the methodology outlined in our paper, we conducted simulations of horizontal gene and TE transfers across three plant species. This was done to examine the aptitude of INTERCHANGE in correctly identifying such HT events without displaying bias. The results from the simulation unequivocally highlight INTERCHANGE's proficiency in detecting both HGT and HTT. To further strengthen these simulation results, as you recommended, we used INTERCHANGE to examine a known recent case of functional gene transfer, which took place from Epichloe to Thynopirum elongatum. A new paragraph detailing this additional analysis has been incorporated into the manuscript. This provides concrete evidence of INTERCHANGE's versatility and precision in dissecting horizontal gene transfers (see page 7 line 3 to 16)

Page 7, line 10. A description of evidence for the historical distribution of these species would have been helpful. Are any of them, for instance, recently introduced?

Response : From the information available to us, we have no evidence suggesting that these species are endemic or recently introduced. Nevertheless, based on their biogeography, all of these species are believed to be indigenous to European and Mediterranean areas. We mention this on page 10, lines 14 to 17.

Supplemental figure 26. These gels of PCR amplicons look unusual. Are they vertical rather than horizontal gels?

Response : The gels are oriented vertically, as depicted in the accompanying photo where the wells are positioned at the top of the gel, suggesting that the migration occurs from top to bottom. To provide additional clarity, we have included this information in the caption of supplementary figures 26 and 27.

Supplemental figure 27. Why do the amplicons of MaCo10 look like the same size? As does MaCo05 in Sup figure 26.

Response : Utilizing the LTRs detected by INTERCHANGE, we designed two distinct sets of primers. These primers were designed by choosing regions that exhibited conservation between the LTRs of the two species involved in the HT, and taking into consideration factors such as melting temperature (tm), GC content, and primer length. Two possibilities explain why there was no observable amplicon size difference of 200 bp, despite our expectations. First, it might be that the migration time was not long enough to reveal this difference, as suggested by the size marker. Alternatively, our estimated amplicon size could be inaccurate, failing to match the actual amplicon size. The primer-designing sequences were derived from assembling short reads of multiple paralogs, resulting in a consensus sequence rather than an authentic genomic copy. This could potentially skew our size estimates. Despite the lack of amplicon size difference, the primary objective of this PCR is to eliminate any chance of contamination by amplifying the transferred elements in two separate individuals. By sequencing the PCR product, we successfully confirmed the presence of the MacO10 element in both Fagus Sylivatica and Hedera helix.

As for MaCo05, we anticipate a nearly identical amplicon size, given that there's only an estimated difference of 80 bp between the two amplicons,

Page 8, line 8. I assume the authors mean “LTR elements” and not just “LTRs”

Response : Yes this is correct. We apologize for the confusion. Throughout the manuscript, we've now substituted 'LTRs' with 'LTR-RTs' to accurately reflect that we are referring to LTR-Retrotransposons."

Page 8, line 22. Was it possible to determine the age of these elements based on LTR similarity? Were the target site duplications identified?

The estimation of transfer age is based on the sequence divergence of the Long Terminal Repeat Retrotransposons (LTR-RTs) between the two species involved in the transfer. The formula used to determine the transfer age is:

Transfer Age = (Sequence Divergence between the LTR-RTs of the species) / (2 * Mutation Rate)

It's worth noting that the application of the molecular clock does come with its own limitations, such as variations in mutation rates across different lineages. The primary aim here is to demonstrate that these transfers are, indeed, ancient events, as indicated by the sequence identity between the transferred LTR-RTs. If the transfers were recent events, we would expect to see extremely high sequence conservation, possibly nearing 100%.

Unfortunately, we were unable to identify TSDs, as the genomes where we detected these horizontal transfers (HTs) are not assembled. The TSDs created by LTR-RTs in plants are typically quite short sequences (4-6 bp). They are not conserved between different elements, as they correspond to duplications of the insertion site that is unique to each insertion. Our tool, INTERCHANGE, is primarily designed to identify conserved regions that result from HTs, hence it is capable of detecting only the transferred elements themselves, not their flanking regions.

Page 9, line 11. 97% is 1.1 million years?
Response : See previous response.

Page 9, line 28. “LTRs-retrotransposons” should be LTR-retrotransposon
Response : Thanks for the comments. We have made the necessary corrections in the manuscript accordingly.

Page 10, line 10. I would imagine that there could be other, technical reasons, for the disparity.

Response : Our analysis of horizontal transfer (HT) in the studied species unequivocally indicates that *Copia* elements are more susceptible to HT compared to the *Gypsy* superfamily. While the two LTR-RT clades differ solely in the order of their coding regions, their sequences exhibit no discernible characteristics that could account for a bias in their characterization. Our pipeline effectively identifies highly conserved sequences derived from HT, including both genes and transposable elements (TEs), as confirmed by our comprehensive test on simulated and real data.

Page 11, line 14. For many reasons, the single element now present may not be at the position of the element when it transferred.

Response : Yes, this is a possibility. INTERCHANGE is designed to detect transferred sequences and not their flanking regions, making it unable to identify the insertion sites of transferred elements. Consequently, determining the original transferred copy becomes very challenging. Once an element is inserted through HT into the recipient genome, it may or may not reactivate, leading to two main scenarios:

1- The element becomes silent after insertion and does not generate new insertions. In this case, finding a single copy of this element in the recipient genome indicates the absence of transposition after transfer.

2- The transferred elements become activated, generating one or several additional copies, followed by the deletion of sibling or master copies. In this scenario, a paralog is retained, which could correspond to the master or a sibling copy.

Differentiating between these two scenarios using the available data is virtually impossible. However, the second scenario is less parsimonious than the first.

In the manuscript, we replace the original sentence "For the HTs where we could identify the direction of HT, we can notice that some *Copia* did not transpose after their transfer (3/8) since they are single copies in the recipient species" with "For the HTs where we could identify the direction of HT, we can observe that some *Copia* elements exist as single copies (3/8), indicating that these elements likely didn't transpose after their transfer."

Page 14, line 19. Is it possible that with relatively low sequencing depth, higher copy number elements would be more difficult to identify relatively rare examples of HT is a high copy sea of elements in both the donor and the recipient species.

Response : There are several reasons to believe that the copy number does not significantly influence the detection of transferred elements with high copy numbers. Initially, the current study presents an example of this; the Gypsy elements (MaGy01) transferred between Acer monspessulanum and Fagus sylvatica is present in 30 copies in F. sylvatica genome. Furthermore, our previous research (El Baidouri et al., 2014) was based on assembled genomes where the majority of transferred elements had a low copy number, even though we examined HTs across all LTR-RTs families, including those with high copy numbers. We did not discuss this observation in that publication, presuming it to be a bias of the studied species. However, in light of this recent study, the propensity of low copy number *Copia* elements for HT can be asserted with great confidence.

**Reviewer #2**: The reviewed manuscript on horizontal transfer "in natura" presents genomic sequencing of 17 wild forest species and a computational pipeline to uncover likely horizontal transfer events in their genome.

The approach is original, more specifically, an improvement on previous procedures used to detect horizontal transfer. The advantage of the presented method is the possibility to use raw sequencing data, as opposed to assembled genomes.

I would like to stress that the described research brings several improvements and novel findings against the current state of affairs:
- the methodologies used so far require prior annotation of the sequences of interest
- the authors identified specific TE families that are more likely to participate in horizontal transfer than other families
- the authors identified lianas and vines as plant species that are more likely to participate in horizontal transfer than other species

Response : We would like to thank you for your valuable understanding and appreciation of the results presented in our current research paper.

Overall, I find the research well planned and carried out, using appropriate arguments and methods. I appreciate the testing on simulated and previously analyzed datasets. However, I identified two problematic issues (labelled as Major Comments below) that should be addressed by the authors. While reading the manuscript I also encountered numerous issues, mostly regarding spelling and grammar which I listed as Minor Comments below.

Major Comments:

1)
The INTERCHANGE pipeline seems to be only introduced by verbal description. In the sake of reproducibility it would be desirable to formalize the pipeline. The minimum acceptable, I think, would be a script with a list of commands and the versions of programs involved. Ideally, however, this would be a formal workflow that could be installed from a repository.

Response : We apologize if our previous explanation was not sufficiently clear. The INTERCHANGE pipeline is hosted on GitHub at 'https://github.com/emaubin/INTERCHANGE'. This is a fully automated pipeline. In the 'Data Availability' section of our manuscript,the link to the pipeline on GitHub is provided. INTERCHANGE only requires two primary inputs: FastQ files of the sequenced species and a table detailing the species names with their corresponding FastQ file paths. A more detailed description of these requirements, installation and user guide can be found on our GitHub page. Once provided with the necessary inputs, INTERCHANGE autonomously carries out a series of operations. It formats the FastQ files and conducts a k-mer analysis, assembles the target regions, and performs the requisite annotations. It also assembles BUSCO genes and test for high similarity to detect HT candidates. The final output from INTERCHANGE is the multifasta of the identified HTs and an easily digestible table. This table includes the names of the implicated species, the names of the scaffolds, sequence identity between the HT candidates, the size of the transferred sequences, and the corresponding annotation (see https://github.com/emaubin/INTERCHANGE)
2)
As opposed to genes, in case of LTR-TEs the sequence may exist in many copies. It seems the authors have not accounted for this when carrying out the simulated sequence tests. They also mention several copies identified on page 11.
I am concerned that the two criteria tested in INTERCHANGE, namely phylogenetic congruence and patchiness could be influenced by this. In the case of congruence, could the identified locus be a subsequent transposition of a TE, which also has a congruent copy?
In case of patchiness, could further transposition and repeat deletions contribute to observed patchiness?

Response : We acknowledge that inferring HTs poses a significant challenge, and confirming the HT hypothesis amidst other plausible alternative hypotheses can be intricate. Nevertheless, our study provides several robust arguments that strongly support the HT hypothesis. We also agree that the presence of multiple paralogs could influence HT inference. This issue is observed when comparing closely related species or highly conserved and slowly evolved genes that have homologs across a large number of taxa, which is not the case for the LTR-RTs we identified in this study.

Plant genomes are highly dynamic compared to animal genomes, leading to the rapid evolution of intergenic sequences, particularly TEs, due to the absence of selective pressure, even among closely related plant species. Consequently, TE lineages diverge at the nucleotide sequence level to such an extent that nucleotide alignment becomes impossible between TEs of divergent plant species. In our current study, the identified LTR-RT elements display remarkable conservation, even across highly divergent plant groups like monocots and dicots, which diverged approximately 150 million years ago. Surprisingly, these elements often exhibit higher sequence similarity than conserved, slowly evolving genes. Such a level of conservation cannot be easily explained by alternative hypotheses such as lineage sorting or gain-loss events, as they fail to account for the high conservation of non-genic sequences over several kilobases (4-5 kbp).

To further bolster the HT hypothesis, we performed an additional analysis utilizing RANGER-TDL software, which tests different scenarios such as gain/loss, duplications, and transfers (see our response to your comment about multiple transfers). This examination clearly confirmed the existence of HTs and rejected alternative hypotheses. Additionally, we validated the reliability of our methodology using the INTERCHANGE test on previously well-known HT cases with elements present in multiple copies in the donor and recipient genomes. This comprehensive evaluation successfully identified the elements involved in HTs, regardless of their copy number. Consequently, this validation further reinforces the credibility of the identified HT events in our study.

The combination of high sequence conservation of non-genic sequences, phylogenetic incongruence, and patchy distribution collectively supports the reported HTs.

Minor Comments:

p2 l1
"especially those of the Ivan and Ale lineages.' <- Ivana?

Response : Thank you for pointing out this error. We have modified the manuscript by replacing the name "Ivan" with "Ivana".

p2 l28
"Host-parasite interactions has been shown to promote HTs" <- have been

Response : Thank you for highlighting this. We correct the sentence in the new version of the manuscript.

p3 l26
"INTERCHANGE a new strategy for horizontal transfer" <- INTERCHANGE: a new... (or "-")

Response : Thank you for bringing this to our attention. We have added a colon (":") in the title, between 'INTERCHANGE' and 'a new'.

p4 l3
"require the prior genome assembly" <- required prior genome assembly? -> or maybe better: "Existing methods" instead of "Previous methods", since the sentences continue in present tense. In any case, drop the article "the".

Response: Thank you for your suggestion. We have now changed the sentence to 'Existing methods for detecting horizontal transfer (HT), which rely on one or two of the previously mentioned three criteria, require prior genome assembly and annotation.

p4 l8
"identifies similar reads derived from conserved locus between the studied species" <- loci (or at least "a conserved locus")

Response : We apologize for the mistake. we've revised the term "conserved locus" to "conserved genomic loci" for clarity.

p5 l11
"False negative correspond" <- negatives

Response : We apologize for this mistake. We have added the "s" to the word "negative".

p6 l1
"INTERCHNAGE" <- INTERCHANGE

Response : Thanks for reporting the typo error. We have corrected the name 'INTERCHANGE'.

p6 l4
"31 HTs candidates" <- HT candidates

Response : Corrected

p6 l9
"are satisfied" <- were
"meet" <- met
Response : Corrected

p6 l14
"INTERCHANGE detect" <- detected

Response : Corrected

p6 l15
"This include" <- "these include" or "this includes"

Response : Corrected

p6 l16
"prunus" <- use peach, since English equivalents are used for all other species mentioned in the sentence
Response : We agree with your comment and have accordingly changed 'Prunus' to 'peach'.

p6 l28
"and whether some particular genes" <- and TO FIND OUT whether...
"Response: We've revised the beginning of the paragraph for better clarity: 'To deepen our understanding of the biotic interactions that can facilitate HTs in natural ecosystems, and to ascertain whether certain genes or TEs are more prone to HT than others, we selected the Massane forest as our study site. This protected reserve, located in southern France, is a unique European location that earned a UNESCO World Heritage Site designation in July 2021.

p7 l3
"2 sparophitic" <- saprophytic
Response : Corrected

p7 l11
"Illumina short-reads technology" <- short read technology

Response : Corrected

p7 l23 and 24
"belongs to the LTR" <- belong
Response : Corrected

"Notably, both the phylogenetic trees and the patchy distribution of these transferred TEs in 400 plant species point to other possible HT of the same elements between multiple plant species, suggesting that these elements may have undergone multiple HTs during their evolution." <- I can't help wondering whether too many other species containing the HT sequence, even if in a patchy distribution, does not support an alternative explanation such as occasional loss of a conserved sequence. Also, could the authors name the most important sequences with multiple-species HTs and maybe provide a complete list as supplement?

Response : To discern between occasional loss and horizontal transfer scenarios, we run the RANGER-DTL program (Mukul S et al 2018). This program leverages the computational approach of Duplication-Transfer-Loss (DTL) reconciliation. This probabilistic models use scalable, parsimony-based methods to comprehend gene evolution. This is achieved by comparing gene trees with species trees to identify duplication, transfer, and loss events. The outcomes of this analysis have conclusively confirmed both the HT events we described, as well as the presence of multiple other HTs events of these elements between other plant species. As you suggested, we have now included the list of these multi-species HTs as Supplemental Table 8 in the manuscript, and made appropriate references to it. Additionally, the corresponding annotated trees have been uploaded as supporting information.

p9 l21
"their respective geographic distributions overlaps" <- overlap
Response : Corrected

p10 l11
"belongs to Ale and Ivana lineage" <- belong to Ale and Ivana lineages

Response : Corrected

p10 l27
"in *Copia* lineages frequencies" <- lineage
Response : Corrected

p10 l31PID
"On average Ale accounted for" <- "On average," <- missing comma
Response : Corrected

p13
"This needs to be tested on a larger sampling of liana species. It is also possible that the tree-to-tree HTs that we identified between beech (F. sylvatica), ash (F. excelsior) or alder (A. glutinosa) could be mediated by other, yet not sequenced, climbing plant species." <- Perhaps the abilities of root system contacts between trees of different species could also contribute?

Response : Indeed, the potential of root system contacts between trees of different species contributing to this phenomenon is an intriguing possibility. However, we currently lack data that could either affirm or reject this hypothesis. This situation is similar to our hypothesis that other climbing plant species may mediate HTs between trees. This is another area of research that we have not yet explored.

p14 l4
"belong mainly to the Ivana and Ale lineage and are low copy numbers" <- belong mainly to the low copy number Ivana and Ale lineages?
Response : Corrected

p14 l15
"The silenced TEs would not be candidate for HT" <- candidates
Response : Corrected

p14 l26
"some lianas species" <- some liana species
Response : Corrected
p14 l31
"Materiel and methods" <- Material
Response : Corrected
p16 l21
"Once identical k-mer" <- kmers
Response : Corrected
p16 l22
"overlapping k-mer" <- kmers
Response : Corrected
p19 l17
"Estimation of copy LTRs number in unassembled genomes" <- Estimation of LTR(-retrotransposon?) copy number(s?) in unassembled genomes
Response : Corrected
p20 l3
"Phylogenetic tree of *Copia* lineages" <- *Copia* (capitalize and italicize)
Response : Corrected
p20
"Multifasta sequences of 12 transferred LTRS (MaCo1 to MaCo12 and MaGy01) are available on this link. PCR product sequences and multiple
alignment of each of the 12 HTs are available on the following link (http://gamay.univ-
perp.fr/~moaine/PCR/)." <- this should be made more permanent, why not include it as a supplement?

Response: Thank you for your suggestion. We have now uploaded these data as supporting informations (see supporting information 2 and 3).

**Have all data underlying the figures and results presented in the manuscript been provided?**
Large-scale datasets should be made available via a public repository as described in the *PLOS Genetics* [data availability policy](http://journals.plos.org/plosgenetics/s/data-availability), and numerical data that underlies graphs or summary statistics should be provided in spreadsheet form as supporting information.

Reviewer #1: Yes

Reviewer #2: Yes

PLOS authors have the option to publish the peer review history of their article ([what does this mean?](_blank)). If published, this will include your full peer review and any attached files.


If you choose “no”, your identity will remain anonymous but your review may still be made public.


**Do you want your identity to be public for this peer review?** For information about this choice, including consent withdrawal, please see our [Privacy Policy](https://www.plos.org/privacy-policy).

Reviewer #1: No

Reviewer #2: **Yes:** Matej Lexa
